# Acknowledgment to Reviewers of *Cancers* in 2021

**DOI:** 10.3390/cancers14030734

**Published:** 2022-01-30

**Authors:** Cancers Editorial Office

**Affiliations:** MDPI AG, St. Alban-Anlage 66, 4052 Basel, Switzerland

Rigorous peer-reviews are the basis of high-quality academic publishing. Thanks to the great efforts of our reviewers, *Cancers* was able to maintain its standards for the high quality of its published papers. Thanks to the contribution of our reviewers, in 2021, the median time to first decision was 18.5 days and the median time to publication was 40 days. The editors would like to extend their gratitude and recognition to the following reviewers for their precious time and dedication, regardless of whether the papers they reviewed were finally published:
Abal, MiguelLewis-Smith, HelenaAbate, GiuliaLeyva, KathrynAbbara, AliLezot, FredericAbboud, TammamLi, BoAbdović, SlavenLi, ChengleiAbe, TatsuyaLi, Chi KongAbed, HasanLi, Chien-FengAbenavoli, LudovicoLi, ChunyingAblain, JulienLi, FangfeiAboubakar, FrankLi, FengzhiAbraham, DietmarLi, FuyangAbrantes, Ana MargaridaLi, Guo-MinAbu Eid, RashaLi, HanAbuhelwa, AhmadLi, HongAbuja, PeterLi, HuiAbusamra, DinaLi, JianfengAceves, CarmenLi, Jian-JianAchreja, AbhinavLi, JiongAcker, GülizLi, MinglunAcquaviva, ClaireLi, QinAcri, GiuseppeLi, Ruei-NianAcs, BalazsLi, Shengwen CalvinAdamczewski, ZbigniewLi, ShizhaoAdamczyk-Grochala, JagodaLi, TaoranAdami, Guy R.Li, WeimingAdams, Brian D.Li, WeizhongAdams, TayloriaLi, XiaohongAdamska, AleksandraLi, YaminAdekoya, Timothy O.Li, YangAdhikari, ManishLi, Yi-jiangAdighibe, OmanmaLi, YingyingAditya, AnushaLi, YongAdukauskienė, DaliaLi, YvonneAdunyah, Samuel E.Li, ZheqiAfelik, SolomonLiagre, BertrandAfonso, PhilippeLiang, ChaoAfonso, RicardoLianos, Georgios D.Agarwal, NeerajLiao, ChenghengAgarwal, SupreetLiao, Guo ShiouAgbarya, AbedLiao, PengAger, AnnLiao, Yung-FengAgerbæk, Mette Ø.Liauw, WinstonAggarwal, VaishaliLibby, GillianAgnieszka, GizakLiberini, VirginiaAgnoletto, ChiaraLibra, MassimoAgostinelli, ClaudioLicht, ThomasAgostini, FrancescoLichtenegger, FelixAgostini, MarcoLicinia, Gouveia ZeliaAgresta, FerdinandoLiebal, UlfAguayo, FranciscoLiersch, TorstenAgudo, JudithLiesveld, JaneAguilar-Company, JuanLifante, Jean-ChristopheAguilera, Nadine S.Lignier, BaptisteAhluwalia, AmritaLikhatskaya, Galina N.Ahluwalia, PankajLim, BoraAhmad, AliLim, KianhuatAhmad, FahimLim, Sang MooAhmad, JavedLim, Tingsen BensonAhmed, KhalilLim, Yu JinAhmed, ZeeshanLimoli, CharlesAhn, KwangLin, Chang-ShenAhn, Seong BeomLin, Chiao-WenAigner, AchimLin, Chien-MinAithal, AbhijitLin, ChingyuAkagi, KiwamuLin, Chun-ShuAkam, Eman A.Lin, EricAkarca, Ayse U.Lin, Huey-JenAkiba, JunLin, Jer-AnAkingboye, AkinfemiLin, Jin-DingAkos, NagyLin, Kuo-ShyanAl Moustafa, Ala-EddinLin, Liang TingAl-Abdulla, RubaLin, Peter P.Alaggio, RitaLin, Po-HanAlaimo, AlessandroLin, Ruo-KaiAlakel, NaelLin, Shi-MingAlarcón Fortepiani, María LourdesLin, Shu FuAlavi, AbassLin, Shu-ChunAlbano, EmanueleLin, Tzu-PingAlbers, PeterLin, Wey RanAlbert, GrinshpunLin, YanAlbini, AdrianaLin, Yen-HungAlcantara, MarionLin, Yu-ChingAlcindor, ThierryLin, Yueh-ChienAldieri, ElisabettaLinares Gómez, MaríaAldrighetti, Luca Antonio M.Linares, Laetitia K.Al-Dujaili, EmadLindenmann, JörgAleem, EimanLinder, BenediktAleman, Berthe M. P.Lindner, KirstenAlesi, Lauren R.Lindner, LarsAlessandro, TafuriLindquist, Jonathan A.Alexander, H. DenisLink, Karl-HeinrichAlexandraki, KrystalleniaLinnebacher, MichaelAlexandre, JérômeLinver, MichaelAlexandre, JoachimLio, Chan-Wang JerryAlexandrova, ElenaLiontos, MichalisAlexa-Stratulat, TeodoraLiotta, Lance A.Alfieri, SergioLipińska, BarbaraAl-Ghabkari, AbdulhameedLipiński, Piotr F. J.Algohary, AhmadLipkowitz, StanleyAli, AimanLiposits, GáborAli, Syed AzmalLisitsyn, Nikolai A.Alic, LejlaLisowska, Katarzyna M.Alifano, MarcoLiss, AndrewAlimandi, MaurizioLita, AdrianAlkhateeb, AbedalrhmanLitwin, Ireneusz BernardAllard-Chamard, HuguesLiu, BolinAllegretti, MatteoLiu, CatherineAllegrucci, CinziaLiu, Chi-MingAllen-Petersen, BrittanyLiu, Chung-JungAllison, Simon J.Liu, DeliAllott, LouisLiu, FengAllouche, MichèleLiu, Jun-JenAl-Mubarak, Bashayer R.Liu, KebinAlonso-Colmenar, Luis M.Liu, KegangAlonso-Gordoa, TeresaLiu, LiAlphonse, Martin PrinceLiu, RuiwuAl-Qurayshi, ZaidLiu, Shi-HeAlsaab, HashemLiu, Shih-JenAlsadeq, AmeeraLiu, TaoAl-Sawaf, OthmanLiu, XiaAlsbeih, GhaziLiu, Yen-LinAlsop, KathrynLiu, Yu-PengAlthouse, AndrewLiu, Yu-YinAltman, Brian J.Liu, Zhi-jie (Jason)Altomare, Deborah A.Liu, ZhongweiAltomonte, JenniferLivi, LorenzoAlunni-Fabbroni, MariannaLivovsky, Dan M.Alvarado-Kristensson, MariaLlanos Casanova, MariaAlvarez Cubero, María JesúsLleonart, Matilde E.Álvarez-Mercado, Ana I.Llop, EstherÁlvarez-Sola, GloriaLlop-Guevara, AlbaÁlvaro, TomásLlorente-Cortes, VicentaAlves Da Costa, CristineLlueca, AntoniAlves, FranciscoLo Nigro, LucaAlves, Marco G.Lo Presti, ElenaAlzhrani, Rami M.Lo Sardo, FedericaAmantini, ConsueloLo, ChiaoAmaraL, TeresaLo, ChristopherAmat, RamonLo, Kwok WaiAmbriovich Ristov, AndrejaLo, SimonAmbros, PeterLobel, LiorAmbrosini, ValentinaLobo, JoaoAmbrosino, FabrizioLocatelli, Marzia AdeliaAmbs, StefanLöck, SteffenAmelio, IvanoLockman, Paul R.Amicone, LauraLoehrer, Patrick J.Amir, SabetLoftås, Per I.Ammendola, MicheleLoginov, Dmitriy S.Ammirati, MarioLoh, Xian JunAmornyotin, SomchaiLohmann, DietmarAmoruso, GiuseppeLohmann, PhilippAmos, Richard A.Lohrish, CarolineAmouroux, MarineLoizidou, Maria A.Amrani, AbdelazizLok, BenjaminAmrutkar, ManojLok, Hiu ChuenAmzar, DanielaLokshin, Anna E.Anand, KartikLomas, OliverAnaniev, JulianLombardi, AngelaAnastasiadou, EleniLombardi, Celestino P.Anatskaya, Olga V.Lombardi, GiuseppeAndersen, Thomas LevinLombardi, TommasoAnderson, Nigel J.Lonardo, AmedeoAnderson, Robin L.Lonardo, EnzaAndersson, JanLondei, PaolaAndersson, Mattias K.London, NyallAndersson, YvetteLone, WaseemAndo, KatsuyoshiLong, Chiau MingAndo, KojiLongatto-Filho, AdhemarAndón, Fernando TorresLonghi, MicheleAndré, GörgensLongo, FrancescoAndré, NathalieŁoniewski, IgorAndrea, LeviLoo, Jia MinAndreani, CristinaLooijenga, Leendert H. J.Andreassi, MariaLoomans-Kropp, HolliAndree, KikiLópez, Giselle Y.Andreetti, ClaudioLópez, José I.Andreev, Dmitry E.Lopez, Jose JavierAndreou, DimosthenisLopez, Juana SerranoAndrés-León, EduardoLopez-Beltran, AntonioAndreucci, ElenaLopez-Bueno, RubenAndreuzzi, EvaLopez-Camarillo, CesarAndrew, Angeline S.Lopez-Charcas, OsbaldoAndrews, MilesLópez-Hernández, Luz BereniceAndria, GregorioLopez-Jornet, PiaAndrieu, NadineLopez-Lopez, ElixabetAndritsch, ElisabethLopitz Otsoa, FernandoAndroutsopoulos, GeorgiosLordan, RonanAnel, AlbertoLorenzo, MicheleAngalakurthi, Siva KrishnaLőrincz, KendeAngelini, FrancescoLőrinczy, DénesAngelova, MihaelaLoschi, MichaelAngelucci, CristianaLosco, LuigiAngioni, RobertaLoskog, AngelicaAngulo, JavierLotti, Lavinia VittoriaAnikin, VladimirLou, ChenguangAnile, MarcoLouhelainen, JariAnken, RalfLoveson, Katie F.Annabi, BorhaneLovšin, NikaAnnunziata, Christina MessineoLozada Nur, FrancinaAnsa, BenjaminLozano, ElisaAnsarin, MohssenLozano-Calderon, SantiagoAnsems, MarleenLozano-Paniagua, DavidAnthony, DonaldLu, Chia-FengAntić, ŽeljkoLu, Chi-YuAntica, MariastefaniaLu, JianfengAntón, AntonioLu, LingengAntonelli, AlessandroLu, Long-ShengAntonelli, RobertaLu, TaoAntonuzzo, LorenzoLu, XinAnvar, ZahraLu, Yen-ShenApostolidis, LeonidasLu, Yong-ChenAppetecchia, MarialuisaLuchinat, ClaudioAppolloni, IreneLuchini, ClaudioAquilanti, Elisa A.Lucia, FrançoisAragaki, MasatoLucioni, MarcoAragon-Ching, Jeanny B.Lucotti, SerenaArai, AyakoLuddy, Kimberly A.Arai, YasuakiLudovini, ViennaAranda, FernandoLudvigsen, MajaArasanz Esteban, HugoLudvikova, MarieAraujo, ArturoLudwig, HeinzAraújo-Gomes, NunoLudwig, MichaelArbajian, ElsaLueong, Smiths S.Arbitrio, MariamenaLukas, Rimas V.Arcovito, AlessandroLulla, AmritiArdizzoni, AndreaLulli, ValentinaArechederra, MaríaLuminari, StefanoAregger, MichaelLun, XiaokangArendowski, AdrianLundgren, Catharina IhreArendt, Lisa M.Lundqvist, AndreasArezzo, AlbertoLunov, OlegArgentiero, AntonellaLuo, DanmengArguelles, SandroLuo, QunArians, NathalieLupiáñez, José A.Aricò, EleonoraLupu, MihaiAriel, AmiramLurje, IsabellaArif, TasleemLuscinskas, Francis William (Bill)Ariga, KatsuhikoLustig, ArthurArima, YoshimiŁuszczki, JarogniewArmant, OlivierLuxton, G. W. GantArmengol, CarolinaLuyt, LenArmengot-Carbó, MiquelLyng, FionaArmocida, DanieleLynge, ElsebethArmstrong, Amy E.Lyros, OrestisArmstrong, NicolaLyu, HuiArnaud, JacquelLyu, RuituArndt, VolkerM′kacher, RadhiaArnold, DirkMa, YafengArosio, DanielaMaccari, AlbertoArribas, JoaquínMaccio, AntonioArruga, FrancescaMace, PeterArsenijevic, TatjanaMacek Jílková, ZuzanaArtemaki, Pinelopi I.Macerola, ElisabettaArtico, MarcoMachairas, NikolaosArul, MichaelMachens, AndreasArús, CarlesMacias, Rocio I. RodriguezArvigo, MaricaMaciej, CieslaAryal, Uma K.Maciejewski, RyszardAsa, Sylvia L.Mack, Elisabeth Karin MariaAsadpour, VahidMacLeod, NicholasAsghar, Muhammad YasirMaçôas, ErmelindaAsher, NethanelMacurek, LiborAshihara, EishiMadamsetty, Vijay SagarAshkarran, AliakbarMaddeboina, KrishnaiahAshton, JohnMader, RobertAshton, NicholasMader, SylvieAspatwar, AshokMadhankumar, Achuthamangalam B.Assaf, ChalidMadhukar, BurraAssié, Jean-BaptisteMadka, VenkateshwarAssy, NimerMadkour, AichaAsti, MattiaMaeda, KiyoshiAstigarraga, ItziarMaes, EvelyneAstl, JaromirMaestro, RobertaAtkins, MardelleMaffini, EnricoAtrash, ShebliMagagnoli, FedericaAttarbaschi, AndisheMaggi, EnricoAtteritano, MarcoMaggi, Leonard B.Attilio, ParisiMaggialetti, NicolaAttique, MuhammadMaggiolini, MarcelloAttwa, Mohamed W.Magherini, FrancescaAu, LewisMagkouta, SophiaAuberger, PatrickMaglietta, GiuseppeAudet-Walsh, ÉtienneMagnaldo, ThierryAudrius, DulskasMagnani, CorradoAugusto, Danillo GardenalMagnes, TeresaAujayeb, AvinashMagnoni, FrancescaAuliac, Jean BernardMagrini, Stefano MariaAureli, AnnaMahadevan, DarukaAurer, IgorMahesh, KasthuriAurrand-Lions, MichelMahmood, NiazAustin, ShaneMahran, AmrAu-Yeung, George H.Maia, CláudioAuzmendi-Iriarte, JaioneMaida, IvanaAvanaki, KamranMaida, PietroAvanzo, MicheleMaina, FlavioAvgeris, MargaritisMaiorano, DomenicoAvila, MatiasMais, ValerioAvnet, SofiaMaisano, DomenicoAvni, DoritMaita, HiroshiAvni, DrorMajchrzak-Celińska, AleksandraAvolio, Alfonso W.Majem, BlancaAvouac, JeromeMajumder, SamarpanAwada, GilMajumder, SarmilaAyuso, José MaríaMak, Nai-KiAzevedo, Maria ManuelMakena, Monish RamAziz, KhaledMakimoto, AtsushiAzizian, AzadehMakishima, HirokazuAzuma, MizukiMakuch-Kocka, AnnaBaba, TsukasaMakvandi, PooyanBabaian, ArtemMalaguarnera, MicheleBaburina, YuliaMalapelle, UmbertoBacher, UlrikeMalara, NataliaBachet, Jean-BaptisteMalavasi, FabioBacigalupo, AndreaMalek, AnastasiaBäcker, DanielMalek, KamillaBadal, SimoneMaleki, ZahraBadescu, Minerva CodrutaMalgor, RamiroBadiola, IkerMałkiewicz, BartoszBaffy, GyorgyMallamaci, RosannaBagby, StefanMallio, Carlo AugustoBagnasco, FrancescaMalpeli, GiorgioBahcecioglu, GokhanMalvezzi, MatteoBahmad, HishamMamessier, EmilieBai, Li-PingMamidi, MuraliBaig, Mohammad HassanMamouni, KenzaBaik, Ku YounMan, FrancisBailey-Whyte, Maeve LouiseManapov, FarkhadBailleul, JustineMancarella, CaterinaBaindara, PiyushMancini, FrancescaBaines, Joel D.Mancini, MaicolBaisi, AlessandroMancini, MariangelaBajor, MalgorzataManconi, AndreaBaker, Darren J.Mancuso, SalvatriceBakht, MartinManda, GinaBalaban, Daniel VasileManem, VenkataBalana, CarmenManes, SantosBalasubramanian, BrindaManet, EvelyneBalayssac, DavidManfredi, SylvainBalázs, MargitMangieri, DomenicaBaldanzi, GianlucaMangla, AnkitBaldari, SergioMango, LucioBalentova, SonaMangone, LuciaBalestri, FrancescoMani, SridharBalgoma, DavidManirujjaman, M.Ballerini, PatriziaMann, Karen M.Balogh, AndreaManneh, RayBaltanás, Fernando C.Mannelli, FrancescoBamdad, CynthiaManning, Amity L.Bandapalli, Obul ReddyMannini, AntonellaBanday, RoufManolopoulos, SpyrosBando, EtsuroManousaki, DespoinaBanerjee, DebabrataManova, VasilissaBaniahmad, AriaMansi, RosalbaBanik, AnnaMansier, OlivierBanito, AnaMantini, GiovannaBano, ShaziaMantovani, FiammaBañuls-Roca, JoséManzo-Merino, JoaquinBao, AndeManzotti, GloriaBar, Eli E.Mapelli, PaolaBarabutis, NektariosMapuskar, Kranti A.Baratta, RobertoMarampon, FrancescoBarauskas, GiedriusMarasco, GiovanniBarb, AdamMarc, JanjaBarbagallo, CristinaMarçais, AntoineBarbara, BreznikMarcato, PaolaBarberio, ManuelMarchal, FrédéricBarbu, Sorin T.Marchand, TonyBarbui, TizianoMarche, Patrice N.Bardella, ChiaraMarchesi, FedericaBarderas, RodrigoMarchetti, LucaBar-Haim, ErezMarchetti, MarcoBarisic, KarmelaMarchica, ValentinaBaritaki, StavroulaMarchini, CristinaBarker, Holly E.Marco, ArdigoBarlier, AnneMarcondes Lerario, AntonioBarnaba, VincenzoMarconi, AlessandraBarnas, EdytaMarconi, GiovanniBarnoud, ThibautMarcos Gragera, RafaelBarok, MárkMarcu, LoredanaBarone, BiagioMarculescu, RodrigBarr, MartinMarcus, Hani J.Barragán, IsabelMargenthaler, Julie A.Barreiro, EstherMarí Alexandre, JosepBarrero, María JoséMaria Carmela, Bonaccorsi Di PattiBarresi, ValeriaMaría Huerta, JoséBarresi, VincenzaMarian, BrigitteBarret, MaximilienMariani-Costantini, RenatoBarrott, Jared J.Marignani, PaolaBarsyte-Lovejoy, DaliaMarignol, LaureBartholomä, Mark DanielMarimuthu, ParthibanBartl, ThomasMarin, OskarBartlett, Edmund K.Marinas-Pardo, Luis AntonioBartlett, Nancy L.Marini, CeciliaBartoletti, MicheleMarini, Herbert RyanBartosinska, JoannaMarinko, TanjaBartosz, GrzegorzMarino, FrancaBartůňková, JiřinaMarino, MariaBartzsch, StefanMario, BignardiBarui, SugataMariotti, Francesca RomanaBarwe, Sonali P.Mariotti, StefanoBarwick, Benjamin G.Marjanski, TomaszBasadonna, GiacomoMark, Tomer M.Basak, DebasishMarkiewicz, AleksandraBasbous, JihaneMarkiewicz, AnnaBaschant, UlrikeMärkl, BrunoBaselet, BjornMarkopoulos, Georgios S.Basolo, FulvioMarkova, SvetlanaBassani-Sternberg, MichalMarko-Vargä, GyörgyBasset-Séguin, NicoleMarkowska, AnnaBassi, Pierfrancesco FrancescoMarks, Daniel L.Basu, ReetobrataMarks, LiannaBatalik, LadislavMarmorino, FedericaBathula, Chandra SekharMarodon, GillesBatie, MichaelMarongiu, FrancescoBatina, NikolaMarotz, JörgBatish, MonaMarques, M. MatildeBattistelli, CeciliaMarquez, JoanaBauchet, LucMarra, FabioBauckneht, MatteoMarra, PaoloBaucum, Anthony J.Marsh, Deborah J.Bauer, JessicaMarshall, LindsayBäuerle, AlexanderMartell, KevinBaujat, BertrandMartelli, Alberto MariaBauman, GlennMartelli, Maria PaolaBaumann, TychoMarti, Rosa M.Baumert, Brigitta G.Marti-Garcia, CeliaBautista, FranciscoMartin, AlejandroBaxevanis, Constantin N.Martin, DanielBaxter, EvaMartin, Elizabeth M.Bayascas, Jose R.Martin, MichèleBayliss, RichardMartin, Robert C. G.Bazzichetto, ChiaraMartin, Simon S.Beato, MiguelMartin, TraceyBeauregard, Jean-MathieuMartina, EmanuelaBeaven, Anne W.Martínez Beamonte, RobertoBechmann, NicoleMartínez López, JoaquínBeck, BenjaminMartinez Macias, Maria IsabelBeck, MarcusMartinez Usatorre, AmaiaBeck, William T.Martínez, ConstantinoBecker, Aline P.Martínez, LidiaBecker, JürgenMartinez, MagalyBednarsch, JanMartinez-Fernandez, LaraBedogni, BarbaraMartínez-Galiano, Juan MiguelBeebe, StephenMartínez-López, JoaquínBeenakker, Jan-WillemMartínez-Pérez, CarlosBeer, David G.Martínez-Soler, FinaBeer, MeinradMartinez-Tapia, ClaudiaBegnaud, Abbie L.Martinez-Useros, JavierBehling, FelixMartinez-Zubiaurre, InigoBehrens, KiraMartin-Hirsch, PierreBeig, NihaMartinho, OlgaBeinat, CorinneMartini, AndreaBekiaris, VasileiosMartini, MaurizioBekkers, Ruud L. M.Martini, VeronicaBélanger, KasandraMartins, José A.Belfiore, AntoninoMartins, NatáliaBelickova, MonikaMartín-Sánchez, EsperanzaBelka, ClausMartins-Neves, Sara R.Bellamri, MadjdaMartín-Vasallo, PabloBelli, Maria LuisaMartynowicz, HelenaBelli, MauroMaruoka, YasuhiroBellini, Maria IreneMarusyk, AndriyBello, Ibrahim OlajideMaruyama, TesshoBelmonte-Beitia, JuanMarvaso, GiuliaBelting, MattiasMarverti, GaetanoBenajiba, LinaMarx, AlexanderBen-Chetrit, NirMary, DidierBendas, GerdMasamune, AtsushiBen-David, YaacovMascagni, DomenicoBenelli, RobertoMascarella, MarcoBenesch, MatthewMascheroni, PietroBenevene, PaulaMaschmeyer, GeorgBenistant, ChristineMascitti, MarcoBennani-Baïti, BarbaraMascolo, MassimoBennardo, LuigiMasedu, FrancescoBennouna, JaafarMasel, Eva KatharinaBensadoun, René-JeanMaslowski, KendleBen-Salem, SalmaMason, RalphBentel, Jacqueline M.Mason, Warren P.Bentivegna, AngelaMasqué-Soler, NeusBenzaquen, JonathanMassari, FrancescoBera, AmitMassarut, SamueleBerardi, Anna ConcettaMassimino, MauraBerasain, CarmenMassone, CesareBerdel, Wolfgang E.Massotte, DominiqueBerdyshev, Evgeny V.Massoumi, RaminBerentsen, SigbjørnMasszi, AndrasBeretta, Giovanni L.Mastrangeli, MassimoBerezovski, Maxim V.Mastrangelo, StefanoBergamini, CarloMastronuzzi, AngelaBergamini, ChristianMasuda, KiyoshiBergandi, LoredanaMasuda, MariBergantini, LauraMasuda, NorikazuBerger, Adam C.Masuda, TakaakiBerger, ClaireMasuda, TakeshiBerger, MassimoMasui, KentaBerkowitz, MichaelaMasuzaki, RyotaBernardi, SimonaMatei, IrinaBernardini, GiovanniMateos, Pilar FernándezBernhardt, PeterMaterka, AndrzejBernstein, Lori J.Matesic, LidiaBerrino, LiberatoMatet, AlexandreBerselli, MattiaMatheu, AnderBerthold, FrankMathew, StephenBerthon, Annabel S.Mathieu, DavidBertolini, FrancescoMathieu, Marie-ChristineBertoni, FrancescoMathieu, VéroniqueBertozzi, SerenaMatiatou, MariaBerveiller, PaulMato, EugeniaBerzins, StuartMatozaki, TakashiBesic, NikolaMatrisian, Lynn M.Besse, LenkaMatsubara, SaburoBesson, CarolineMatsuda, YokoBetancourt, LazaroMatsui, YoshiyukiBetés, Maite TeresaMatsuki, TakahiroBette, MichaelMatsukuma, SatoshiBetz-Stablein, BrigidMatsumoto, KazumasaBeuriat, Pierre-AurélienMatsumoto, NaokiBevilacqua, AlessandroMatsumoto, SeiichiBewersdorf, Jan PhilippMatsumoto, YoshihisaBeylot-Barry, MarieMatsuo, YukikoBeyreuther, ElkeMatsusaki, TakashiBezu, LucilliaMatsuzaki, ShinyaBhagirath, DivyaMatthies, CordulaBharati, SanjayMatthiesen, RuneBhardwaj, RajeshMatthiessen, PeterBhardwaj, VikasMattonen, SarahBhargava, RheaMatulic, MajaBhat, Ambarish P.Matveeva, OlgaBhat, KamakotiMatyjaszek-Matuszek, BeataBhat, RakshaMaugeri, Andrea GiuseppeBhattacharya, AbhisekMaugeri, RosarioBhattacharya, RajatMaughan, Benjamin L.Bhattacharya, SantanuMaura, CalvaniBhattacharya, SomanonMaurea, SimoneBhattacharyya, SohineeMaurer, JochenBhawal, Ujjal KumarMaurer, StefanieBhowmick, Neil A.Mauri, DavideBiamonte, FlaviaMaurizio, AuroraBianchi, AlbertoMaury, Jean MichelBianchi, LorenzoMausolf, Edward J.Bianchi, MarziaMavinkurve-Groothuis, Annelies M. C.Bianchi, SerenaMavrogonatou, EleniBianchini, LaurenceMavroidi, BarbaraBianchini, RodolfoMavros, MichailBianco, Maria RitaMavroudis, DimitriοsBianco, RobertoMavuluri, JayadevBianconi, FortunatoMawhinney, ThomasBiasoli, IreneMaximov, IvanBiassoni, RobertoMay, FelicityBibault, Jean-EmmanuelMayani, HectorBibikova, OlgaMaycotte, PaolaBidoli, EttoreMayer, ArnulfBiedermann, DavidMaynard, Janielle P.Bielawska-Pohl, AleksandraMayo, Samantha JBielawski, KornelMayoh, ChelseaBielinsky, Anja KatrinMazan-Mamczarz, KrystynaBieńkowski, MichałMazurek, Agnieszka M.Biersack, BernhardMazurek, UrszulaBiewald, EvaMazzanti, Chiara MariaBiffo, StefanoMazzei, Maria AntoniettaBiffoni, MarcoMazzola, MicheleBigley, Austin B.Mazzoni, MariaBijlsma, Maarten F.Mazzucchelli, RobertaBikas, AthanasiosMcArdle, StephanieBilbao, DanielMcCaughan, GeoffBilger, AndreaMcCaughan, Geoffrey WilliamBill, MariusMcCaul, James AnthonyBilodeau, MarcMcClurg, UrszulaBilski, JanMcComb, ScottBinotto, GianniMcCullagh, JamesBiondi, Antonio GiuseppeMcdonald, JeffreyBirkeland, Andrew C.McEwen, GayleBirkó, ZsuzsannaMcGowan, EileenBishayee, AnupamMcGrowder, DonovanBishehsari, FarazMcIntosh, Stuart A.Bishnupuri, Kumar S.McKeon, FrankBismar, TarekMcKinstry, Karl KaiBistulfi, GaiaMcLean, KarenBitzer, MichaelMcMullin, Mary FrancesBjörkblom, BennyMcNally, Lacey R.Blache, PhilippeMcNally, OrlaBlack, Joshua C.McReynolds, Lisa J.Blackburn, JessicaMeade, Aidan D.Blandino, GiovanniMèchinaud, Françoise M.Blank, Alan T.Medenwald, DanielBlank, SusanneMedipally, DineshBlank, VolkerMeehan, KatieBlanquart, ChristopheMego, MichalBlattner, ChristineMehrabi, ArianebBlay, Jaen-YvesMehta, AmbereenBleckmann, AnnalenMehta, Gaurav A.Bleeker, Maaike C. G.Mehta, SunaliBlenda, AnnaMehterov, NikolayBloch, WilhelmMei, Kuo-ChingBoada, AramMei, Ya-FangBoakye, DanielMeiburger, Kristen M.Boaventura, PaulaMeignan, MichelBobba, Kondapa NaiduMeiler, JohannesBobiński, MarcinMeiser, JohannesBoccalatte, FrancescoMelcón, GallegoBochtler, TilmannMelendy, ThomasBoehm, KatjaMelenhorst, Jan JosephBogacka, IwonaMelgaço, Juliana G.Bogani, GiorgioMeli, FrancescoBoggio, ElenaMellai, MartaBogucki, JacekMellitzer, GeorgBohmer, DirkMelloni, ElisabettaBoileau, EtienneMelo, Sonia A.Boilève, AliceMelstrom, Kurt A.Boinska, JoannaMeltzer, SebastianBoix, LoretoMena, MarisaBoj, Sylvia F.Menard, ArmelleBojarska-Junak, Agnieszka A.Mendichovszky, IosifBojunga, JoergMenè, PaoloBolam, Kate A.Menéndez-Menéndez, JavierBoldrini, LucaMeng, XiangbingBolm, LouisaMenghi, FrancescaBolomsky, ArnoldMenichetti, LucaBommareddy, PraveenMenke, AndreBommireddy, RamireddyMennerich, DanielaBoms, StefanieMenon, Dinoop RavindranBomsztyk, KarolMensink, Hanneke W.Bonavita, EduardoMercer, ClaireBonetti, AndreaMercke, ClaesBonferoni, Maria CristinaMerkel, SusanneBongiovanni, AlbertoMerli, PietroBongiovanni, MassimoMerola, ElettraBonifacio, MassimilianoMerolla, FrancescoBonin, SerenaMertens, ChristinaBonino, FerruccioMerz, ValeriaBonnet, Pierre-AntoineMesguich, CharlesBonnici, VincenzoMessaritakis, IppokratisBono, HidemasaMessersmith, AmyBonora, ElenaMessiha, HadiBoo, Yong ChoolMessner, FrankaBoon, SiawMesuraca, MariaBoppana, Nithin B.Metere, AlessioBorcherding, Dana C.Metovic, JasnaBorcherding, NicholasMetzen, EricBorg, DavidMetzler, MarkusBorgia, Jeffrey A.Meyer Zu Heringdorf, DagmarBorgna, FrancescaMeyer, Hans-JonasBorisenkov, Mikhail F.Meyer, MoritzBorkowetz, AngelikaMeyer, PeterBorkowska, Edyta MartaMeyers, CraigBornfeld, NorbertMezencev, RomanBornhaeuser, MartinMezi, SilviaBorrelli, PaolaMezõsi, EmeseBørset, MagneMiah, AishaBorson-Chazot, FrançoiseMiao, LinglingBorstnar, SimonaMiceli, VitaleBortner, Carl D.Michael, AgnieszkaBorzi, CristinaMichael, MichaelBorzio, MauroMichaelis, MartinBoschert, VerenaMichalis, EurydikiBose, DebojitMichalsen, AndreasBosi, AlbertoMichaud, KarineBosnjak, MasaMichelacci, Yara M.Bost, FrédéricMichelhaugh, Sharon K.Bostan, MarinelaMichelle, PalmieriBoström, Jan PatrickMichels, JudithBôta, AttilaMichikawa, TakehiroBotella, Luisa MaríaMicke, PatrickBotta, CirinoMiederer, MatthiasBottalico, LucreziaMigliaccio, AntimoBotticelli, AndreaMihara, KeichiroBotton, ThomasMihaylov, PlamenBouchal, PavelMikaelyan, Arsen S.Boucheix, ClaudeMiklikova, SvetlanaBoudreau, Howard E.Miklós, GyöngyBougen-Zhukov, NicolaMikolajczak, RenataBouleuc, CaroleMikolajczyk, RafaelBouley, ReneeMikropoulos, ChristosBouley, RichardMikula, MichalBoulkroun, SheerazedMilanowski, MaciejBoulter, LukeMilczarek, MałgorzataBourdon, EmmanuelMilella, MicheleBourdon, Jean-ChristopheMileo, Anna Maria NiveBousbaa, HassanMiles, LindseyBousquet, CorinneMiletic, HrvojeBousquet, GuilhemMiliauskas, SkaidriusBoussios, StergiosMilic, MarinaBouvier, Anne-MarieMiligi, LuciaBouwman, PeterMilitello, CarmeloBove, AldoMiljković, Miloš D.Bovell, LiselleMiller, Anthony B.Bowen, DeborahMiller, Ian SteuartBowman, CaitlynMiller, John H.Boyer, ThomasMiller, KimberlyBoyle, Glen M.Miller, VandanaBozec, AlexandreMiller, William B.Bożena, Leszczyńska-GorzelakMiller, Wilson H.Bozic, IvanaMillesi, MatthiasBozic, JoskoMills, KenBraathen, LasseMin, IreneBrabek, JanMina, RobertoBracht, Jillian Wilhelmina PaulinaMinafra, LuigiBrady, AdrianMinami, YasunoriBrägelmann, JohannesMinami, YosukeBraicu, CorneliaMinamikawa, TakeoBrakenhielm, EbbaMinamimoto, RyogoBrambilla, DarioMinea, RaduBranca, Jacopo Junio ValerioMinervini, GiovanniBrancaccio, MariaritaMinna, EmanuelaBrancati, NadiaMinnelli, CristinaBrandal, PetterMinucci, AngeloBrandi, NicolòMinutolo, FilippoBrandt, BurkhardMio, CatiaBranzei, DanaMirabilii, SimoneBrasher, Penelope M. A.Miricescu, DanielaBrassart-Pasco, SylvieMirjolet-Didelot, CélineBratashov, Daniil N.Mirlekar, BhalchandraBrattsand, MariaMirzaei, SiroosBraun, FelixMirzayans, RazmikBraun, ThorstenMischkulnig, MarioBrauner, EdoardoMiserocchi, GiacomoBraunstein, Evan M.Mishra, AvanishBrechbuhl, HeatherMishra, ManojBrégier, FrédériqueMishra, RosalinBreier, GeorgMishra, Sandeep KumarBremer, EdwinMiskiewicz, PiotrBrenet, FabienneMiszczyk, JustynaBrennan, DonalMita, KojiBrennan, GregMitani, SeiichiroBrenner, J. ChadMitchell, PaulBrenner, John ChadMitjavila, Mercedes CasanovaBresciani, RobertoMitobe, YuichiBresson-Bepoldin, LaurenceMitola, StefaniaBrézillon, StéphaneMitra, MithunBria, EmilioMitra, RanjanaBrichkina, AnnaMitra, SumeghaBrieger, AngelaMitsogiannis, IraklisBrindley, DavidMitsuishi, YoichiroBrioli, AnnamariaMiura, KouheiBrito, CatarinaMiura, SayakaBroccoli, AlessandroMiyagi, YoheiBrockmeyer, PhillippMiyamoto, HirotakaBrodska, BarboraMiyamoto, IkuyaBrody, Jonathan R.Miyanaga, AkihikoBroekgaarden, MansMiyazaki, MasaruBroggi, GiuseppeMiyazawa, MasaakiBroggini, MassimoMiziak, BarbaraBronte, FabrizioMizukami, TatsujiBrooke, GregMizuno, ShugoBrooks, AllenMo, FeiBrosens, ErwinMo, Lein-RayBrossart, PeterMobasheri, AliBrosseau, Jean-PhilippeMochizuki, SatsukiBrouchet, AnneModerau, NinaBrounais-Le-Royer, BénédicteMoertl, SimoneBrown, JamesMogler, CarolinBrown, Jeffrey W.Mograbi, BahariaBrown, JustinMohammed, AltafBrown, NelsonMohan, MeeraBrown, Ronald B.Mohanty, VakulBrozovic, AnamariaMohapatra, BhopalBrozzetti, StefaniaMohibi, ShakurBrugnoli, FedericaMohl, Jonathon EdwardBrummer, TilmanMöhlendick, BirteBruni, LaiaMohr, ThomasBrunner, MarkusMoia, RiccardoBrunner, MaximilianMojarad, Ehsan NazemalhosseiniBrunner, ThomasMojsilović, Slavko B.Brünnert, DanielaMojumdar, KamalikaBruno, AntoninoMokbel, KefahBruno, FedericoMoldovanu, SimonaBruzzaniti, PlacidoMoles, AnnaBruzzese, FrancescaMoles, Jean-pierreBryant, HelenMolfetta, RosaBuccisano, FrancescoMolica, MatteoBuchler, TomasMolin, DanielBuck, Michael D.Molina, Eric SueroBuckle, AshleyMolina, Thierry JoBuechert, MartinMolina-Cerrillo, JavierBuffi, NicolòmariaMoll, AnnetteBugada, DarioMöller, GabrieleBugalho, Maria JoãoMomčilović, Stefan D.Buijs, Jeroen T.Monacelli, FiammettaBujanda, LuisMonaco, GiovanniBujko, MateuszMoncayo, RoyBukkuri, AnuraagMondal, GoutamBulat, TanjaMondal, TanmoyBulfone, GiampieraMondelli, Mario U.Bulin, Anne-LaureMonsalve, MariaBunce, Chris M.Montalban-Arques, AnaBundschuh, Ralph A.Montalbano, MauroBunevičius, AdomasMontano, NicolaBunin, Greta R.Monteleone, GiovanniBuoncervello, MariaMontemurro, NicolaBurchell, JoyMontorsi, MarcoBurckhardt, ChristophMontrucchio, GiuseppeBurdach, StefanMonzani, FabioBurdak-Rothkamm, SusanneMoon, DominicBurden, RobertaMoon, EjungBure, Irina V.Moon, Sung-KwonBurek, MalgorzataMoore-Carrasco, RodrigoBurger, Michael C.Moormann, AnnBurgett, Anthony W. G.Moosa, Mahdi MuhammadBürglin, Thomas R.Mora, FrancescoBurgos-Aceves, Mario AlbertoMora, JaumeBurkard, Mark E.Morais, MauricioBurnell, MatthewMorais, RuiBurra, PatriziaMorak-Młodawska, BeataBurster, TimoMorales, HumbertoBuscail, LouisMorales, Jose ManuelBusch, JonasMorales, JulioBusch, MaikeMorales, SerafinBusetto, Gian MariaMora-López, LauraBuske, ChristianMorandi, LucaBusse, AntoniaMorani, FedericaBüsselberg, DietrichMoreaux, JeromeBussolati, OvidioMorel, AlainBustoros, MarkMorel, PierreBuszewska-Forajta, MagdalenaMorelli, LucaButler, EboneéMorelli, Maria BeatriceBüttner, ReinhardMorello, SilvanaButturini, ElenaMoreno, Carlos S.Buzdin, AntonMoreno, EstefaníaBuzza, MarkMoreno, J. J.Bychkov, AndreyMoret, FrancescaBye, Alexander P.Moretti, SoniaByrne, HughMorgan, Ethan L.Byung Chang, KimMorgan, MichaelCaballero-Villarraso, JavierMorganti, Alessio GiuseppeCabañas, CarlosMori, MattiaCackowski, FrankMori, YasuhisaCadamuro, MassimilianoMorigi, RitaCadilha, Bruno L.Moris, LisaCadossi, RuggeroMorita, YoshihiroCadoux-Hudson, Thomas A. D.Morito, KurataCadum, EnnioMoritz, Rose K. C.Caers, JoMorizane, RyujiCaetano, JoanaMorjani, HamidCaffo, MariaMorkel, MarkusCafri, GalMoro, FrancescaCagini, LucioMorotti, AlessandroCagnazzo, CelesteMorra, FrancescoCagnin, StefanoMorra, SimoneCai, ChunyuMorrione, AndreaCai, LishengMorris, DonCaimi, PaoloMorrison, JoCairrão, ElisaMorrissey, MariaCalcabrini, AnnaricaMorselli, SimoneCaligo, Maria AdelaideMoscetti, LucaCalò, ValentinaMoser, BernhardCalogero, AntonellaMosqueira, Vanessa Carla FurtadoCaltabiano, RosarioMoss, Esther LouiseCalvaruso, MarcoMotamed, CyrusCalvisi, Diego FrancescoMotea, Edward A.Calvo, Gabriel F.Motegi, AtsushiCamacho-Arroyo, IgnacioMotiam, Ahmed ElCambien, BéatriceMotoi, FuyuhikoCameron, DonaldMotola, DomenicoCameselle-Teijeiro, Jose ManuelMotoyama, SatoruCammarata, FrancescoMoulik, SabyasachiCampa, Carlo CosimoMoulin, AlexandreCampagna, RobertoMouneimne, GhassanCampagne, OliviaMountzios, GiannisCampagnola, Paul J.Moura, David S.Campana, DavideMourtada-Maarabouni, MirnaCampanella, FabioMowday, AlexandraCampbell, KerryMpakali, AnastasiaCampione, SeveroMravec, BorisCampolo, FedericaMuallem, Mustafa ZelalCampolo, MichelaMudryj, MariaCampos, Camila D. MadeiraMueller, MathiasCamps, JordiMuenst, Simone E.Canbay, AliMuether, MichaelCanbay, EmelMuggeo, PaolaCanberk, SuleMuhammad, Jibran SualehCandoni, AnnaMühlebner, AngelikaCanela, Enric I.Mukherjee, SudipCanesin, GiacomoMukhopadhyay, SumanCanevari, SilvanaMulhearn, BenCannarozzo, GiovanniMulholland, EoghanCantin, André M.Müller, Klaus GottlobCantini, GiuliaMuller, LaurentCanu, LetiziaMuller, MandyCañueto, JavierMüller, StefanCanzona, MollieMüller-Decker, KarinCao, KimMun, Seong K.Cao, YihaiMunakata, SatoruCapaccio, PasqualeMunir, KhushbooCapece, DariaMuniraj, NethajiCapeloa, TaniaMunker, ReinholdCapiod, ThierryMunkley, JenniferCapobianco, GiampieroMuñoz Sáez, MiguelCapoluongo, EttoreMunz, ChristianCaponio, Vito Carlo AlbertoMuragaki, YoshihiroCappariello, AlfredoMurata, SoichiroCappelletti, VeraMurata, TakayukiCappello, PaolaMuresan, Mihai StefanCapuano, AlessandraMurotani, KentaCapula, MjriamMurphy, AndrewCarai, AndreaMurphy, BrittanyCaramalho, IrisMurphy, BronaCaramujo, Maria JoséMurray, DavidCaratozzolo, Mariano FrancescoMurray, JayneCarbognin, LuisaMurray, PaulCarbonaro, Luca A.Murthy, DivyaCarbone, AntoninoMurugan, SengottuvelanCarbone, DanielaMuruganandan, SanjeevanCarcereri De Prati, AlessandraMurugesan, KanagavelCarchman, Evie H.Murugesan, SelvasankarCardile, VeneraMuscarella, Lucia AnnaCardillo, GiuseppeMuscolini, MichelaCardobi, NicolòMusi, GennaroCarè, AlessandraMussolin, LaraCare, AndrewMuz, BarbaraCarey, ReneeMuzio, MartaCarlberg, CarstenMuzza, MarinaCarleo, AlfonsoMycielska, MariaCarlisi, DanielaMymryk, JoeCarloni, RiccardoNa, KiyongCarlos, Ana RitaNabiee, RominaCarmen, MejíaNadanaka, SatomiCarneiro, PatríciaNadeau, Jay L.Carnesecchi, JulieNagano, HiroakiCaro, CarlosNagasaka, KazunoriCarotenuto, PietroNagata, YosukeCarpizo, Darren R.Nagelkerke, AnikaCarr, Robert A.Nagler, ArnonCarrà, GiovannaNagrani, RajiniCarrano, AnnaNahain, AbdullahCarraro, FabioNaidu Chitrala, KumaraswamyCarrer, AlessandroNaillat, FlorenceCarrera, PilarNair, Praful R.Carreras, JoaquimNaito, TateakiCarrier, FranceNaito, YutakaCarriero, AlessandroNajahi Missaoui, WidedCarruthers, RossNakagawa, HidewakiCarter, WayneNakagawa, TakuroCaruana, IgnazioNakahara, TomomiCarullo, GabrieleNakai, YousukeCaruntu, AnaNakajima, OsaniuCaruntu, ConstantinNakajima, TakahiroCarus, AndreasNakajima, TakahitoCaruso, GerardoNakamura, NaoyaCarvalho, EdmundNakamura, TakuroCarvalho, JoanaNakamura, YasuhiroCasadio, PaoloNakamura, YoshiyukiCasado, PedroNakamura, YusukeCasalou, CristinaNakanishi, NaohikoCasanova Acebes, MaríaNakano, KenjiCasanova, EmilioNakano, MasahitoCasares, NoeliaNakano, ToshiakiCasas, JosefinaNakashima, SouichiCascini, Giuseppe LucioNakayama, NoriyukiCascio, SandraNakayama, RobertCasey, JenniferNakazawa, TsutomuCasimiro, SandraNallanthighal, SameeraCassano, EnricoNalvarte, IvanCassim, ShamirNami, BabakCastelli, VanessaNamløs, Heidi M.Castelo-Branco, PedroNanda, NehaCastillo-Tong, Dan CacsireNanduri, Siva Lahiri KanthCastro, FláviaNannini, MargheritaCastro-Vega, Luis JaimeNarayana, ShaliniCatchpoole, DanielNardi, MariateresaCatela Ivkovic, TinaNarita, NorihikoCatic, AndreNarkhede, MayurCatino, AnnamariaNaryzhny, Stanislav NCattrini, CarloNascimento, Bruno F. O.Cauli, OmarNaselli, AngeloCaux, ChristopheNash, RobertCava, ClaudiaNashine, SonaliCavaco, DanielaNasillo, VincenzoCavalieri, StefanoNason, GregoryCavallari, EleonoraNass, NorbertCavalloni, GiulianaNassir, FatihaCavazzoni, AndreaNassiri, MehdiCavic, MilenaNatalello, AntoninoCavo, AlessiaNatarajan, SivaramanCayrefourcq, LaureNath, ShubhankarCazacu, IrinaNatkunam, YasodhaCaziuc, AlexandraNatsumeda, ManabuCazzola, EmilianoNaumann, NicoleCea, MicheleNausica, MontaltoCecinati, ValerioNava, VictorCelentano, EgidioNavarria, PierinaCenariu, MihaiNavarro, José-TomásCenni, VittoriaNavran, ArashCepeda, SantiagoNawas, Afshan FathimaCeppi, LorenzoNawaz, Mohd ImtiazCeraline, JocelynNawroth, RomanCerchia, LauraNayak, Jasmir G.Cerchione, ClaudioNayar, ManuCerdán, María-EsperanzaNazaruk, EwaCernuda, RafaelNazio, FrancescaCervera, JoséNeagoe, Ioana BerindanCervigón, RaquelNeagoe, OctavianCerwenka, HerwigNechaev, SergeiCescon, MatteoNeels, OliverCesetti, TizianaNees, MatthiasChabay, PaolaNegoro, HiromitsuChaber, RadosławNegri, Gian LucaChabot, BenoitNegri, RodolfoChacko, Jenu VargheseNeill, Stewart G.Chahwan, RichardNel, IvonneChakrabarti, JayatiNelson, GradyChakraborty, Abhishek A.Nemunaitis, John J.Chakraborty, AnirbanNeophytou, Christiana M.Chakraborty, SayanNephew, Kenneth PChakroborty, DebanjanNerreter, ThomasChallis, Benjamin G.Nervi, ClaraChan, JasonNessim, CarolynChan, Jason Y. K.Neufert, ClemensChan, Karen Kar LoenNeuhaus, JochenChan, Keefe T.Neureiter, DanielChan, Sheng-ChiehNeuwelt, Alexander J.Chan, WaikinNeven, PatrickChandra, JaninNevi, LorenzoChandrasekaran, AkshayaNevzorova, YuliaChang, Ching-JinNewhouse, IanChang, EunjuNey, Douglas E.Chang, Hsueh-WeiNgezahayo, AnacletChang, Jer-HaoNgo, ThuyChang, Julia Yu-FongNguyen, FrédériqueChang, MeichiNguyen, Jane K.Chang, Nan-ShanNguyen, Minhhuyen T.Chang, Shu-ChunNi, WeiChang, Wei-LunNiazi, KhalidChang, Wei-MinNibali, Marco ContiChang, Wen TsanNicolas, EmmanuelleChang, Wen-WeiNicoletti, FerdinandoChang, Yu ChanNicos, MarcinChapman, Paul B.Nicoś, MarcinChardes, ThierryNiculae, DanaCharli-Joseph, Yann VincentNie, DaotaiCharriere, GuillaumeNieland, John Dirk VestergaardChatain, NicolasNielsen, Finn CiliusChatelut, ÉtienneNielsen, KariChatterjee, AniruddhaNieminen, Taina T.Chatterjee, NimratNiemiro, Grace M.Chatterjee, SampurnaNienhüser, HenrikChattopadhyay, Sajal K.Niessner, HeikeChauvie, StephaneNiewald, MarcusChaves, Susana RodriguesNii, TerukiCheema, UmberNiikura, RyotaCheifetz, RonaNijhof, IngerChelakkot, ChaithanyaNijland, MarcelChelikani, PrashenNijnatten, ThiemoChellaiah, MeenaNikas, Ilias P.Chemello, LilianaNikfar, ShekoufehChen, Bang-BinNikitovic, DraganaChen, Chang-HanNikolova, BilianaChen, ChaolingNimura, KeisukeChen, Chien-ChinNing, ShunbinChen, Chih-Shan JasonNirala, Niraj K.Chen, Chi-LongNiscola, PasqualeChen, Chung-HsinNishida, HarutoChen, Chun-HanNishida, HirokoChen, Chun-LiangNishida, ShozoChen, DexiNishida, ToshiroChen, Georgia ZhuoNishida, TsutomuChen, GuanNishikawa, HirokiChen, HanyingNishimoto, KoshiroChen, HaoNishimura, NoriyukiChen, HongweiNishina, HiroshiChen, HongwuNishio, MizuhoChen, HuNishio, ShinChen, HuarongNishizawa, ToshihiroChen, JinbiaoNissinen, Tuuli A.Chen, JingNisticò, Steven PaulChen, Jui-ChiehNitika, NitikaChen, Ke-ChengNitulescu, George MihaiChen, Kuang-ChiNitulescu, MihaiChen, LichingNixon, Daniel W.Chen, LihuaNiza, EnriqueChen, LongwenNjoku, KelechiChen, Mei-ChuanNjor, Sisse HelleChen, MingNoberini, RobertaChen, MingshengNobili, StefaniaChen, MingyiNobis, MaxChen, Pai-ShengNogova, LuciaChen, Rong-JaneNoguera, Nélida InésChen, San-YuanNoh, Jae MyoungChen, Sean Chun ChangNoh, Ji-YoonChen, Shaw-JiNoh, Woo ChulChen, Sheng-HungNonnekens, JulieChen, ShihlungNoonan, DouglasChen, Shin-ChehNoorani, ImranChen, ShukunNoordermeer, SylvieChen, Si-TongNoothalapati, HemanthChen, WeiqiangNorcic, GregorChen, Yann-JangNordström, AndersChen, YifanNordstrom, RobertChen, Yih YuanNoro, RintaroChen, Yih-FungNøst, Therese HaugdahlChen, YuchihNoubissi-Kamdem, FeliciteChen, Yu-ChihNovakova, ZoraChen, ZeyuNovick, DanielaChen, Zhe-shengNowak, DanielChen, ZiguiNowak, Michal S.Cheng, Alfred Sze-LokNowakowska-Zajdel, EwaCheng, Chia-HsiungNtanasis-Stathopoulos, IoannisCheng, ChunchiaNuciforo, PaoloCheng, ChunmingNuhn, PhilippCheng, HenrichNumakura, KazuyukiCheng, HsutangNürnberg, SylviaCheng, Hung-ChiNy, LarsCheng, Jing-JyNylander, KarinCheng, KunNylen, CarolinaCheng, Ming-HueiNyström, AlexanderCheng, Tsu-YaoO′Dwyer, MichaelCheong, Jit KongO′Leary, JohnChepelev, LeonidO′Reilly, SeamusCherdyntseva, Nadezhda V.O′Rourke, ColmCherkasova, OlgaO′Toole, Sharon A.Chernyshev, VasiliyOancea, S. CristinaCherukula, KondareddyObata, YuukiChessa, LucianaOberacker, EvaCheung, Kwok-LeungObermayr, EvaCheung, MartinObi, ShuntaroChevalier, FrançoisObinata, DaisukeChhabra, GaganOchieng, JosiahChi, Hsiang-ChengOcker, MatthiasChi, Kwan HwaOda, TsukasaChia, Jean-SanOddone, EnricoChiang, J. M.Oechsle, KarinChiang, NaijungOehler, RudolfChiang, WeichungOehme, InaChiang, Ying-ChengOerlemans, SimoneChiang, Yi-TingOffer, StevenChiara, María-DoloresOffermann, AnneChiarion-Sileni, VannaÖfner-Velano, DietmarChiavassa, SophieOgawa, TetsuyaChiba, HideyukiOgino, ShujiChicano-Gálvez, EduardoOgino, TakashiChiche, JohannaOgiwara, HideakiChichiarelli, SilviaOgnibene, MarziaChiefari, EusebioOgris, ManfredChien, Chih-YenOh, Eun-TaexChien, Chun-RuOh, Seh-HoonChihara, DaiOh, SekyungChimenz, RobertoOhara, ToshiakiChinen, LudmillaOhashi, ManabuChintala, SreenivasuluOhba, KojiroChiocchetti, AnnalisaOhishi, TomokazuChiofalo, Maria GraziaOhkawa, KazuyoshiChirgwin, John M.Ohkuri, TakayukiChitale, ShalakaOhno, HitoshiChitcholtan, KennyOhnuki, HidetakaChithrani, DevikaOhtsuka, TakashiChittiboina, PrashantOhyama, TetsujiChiu, Ching-FengOikawa, KosukeChiu, Hua-ShengOji, YusukeChiu, IngmingOkabe, SeiichiChiu, Norman H. L.Okada, TomoyoChiuri, Vincenzo EmanueleOkami, KenjiCho, AnnaOkamoto, MarikoCho, ChoifongOki, KenjiCho, Jae HeeOkita, YoshikoCho, JeongheeOklu, RahmiCho, MayOkonechnikov, KonstantinCho, Shih-FengOkonogi, NoriyukiCho, Young-YounOkosun, JessicaChodick, GabrielOksenych, ValentynChoi, ChanghoonOkubo, HironaoChoi, Hyo GeunOkumura, ToshiyukiChoi, JiyeonOkumura, YasuhiroChoi, Min-chulOkuno, ScottChoi, Siu WaiOkusaka, TakujiChoi, YoungOlaru, Octavian TudorelChong, GeoffOlaya Abril, AlfonsoChong, Irene Yu-ShingOlbryt, MagdalenaChong, YosepOle Schmidt, NilsChou, Angela S.Olin, MichaelChou, Cheng-YangOliveira, Maria CristinaChou, Chih-HungOliveira, Sonia M.Chou, Chih-MingOliver, LisaChou, Wen-ChienOllauri-Ibáñez, ClaudiaChoudhury, Parichoy PalOlmo, AlbertoChrétien, Anne-SophieOlsen, MajaChristen, ThomasOlsson, Lars E.Christian, SelingerOmerovic, JasminkaChristiansen, HansOmilusik, Kyla D.Christie, MichaelOnadim, ZerrinChristmann, MarkusOno, YoshihiroChristopher, LaRoccaOnoda, NaoyoshiChristopoulos, PetrosOpalińska, MartaChrzanowska, AlicjaOpländer, ChristianChu, Cheng-YingOprea - Lager, DanielaChu, Ching-LiangOpyrchal, MateuszChu, Wai KitOratz, RuthChu, XiangpingOrchard, SandraChuang, Shih-SungÖrd, TiitChuang, Show-MeiÖrd, Tönis A.Chuang, Wan-LongOrdonez-Moran, PalomaChulián, SalvadorOrefice, NicolaChun, SejongOrimo, AkiraChun, Yang-SookOrion, ItzhakChung, Byung MinOrlandella, Francesca MariaChung, Chi-LiOrlandi, RosariaChung, Ching-HuOrlova, AnnaChung, ChristineOrme, JacobChung, Felicia Fei-LeiOrsi, NicChung, Jae HoonOrtiz, RaulChung, Joon-YongOrtiz-Cuaran, SandraChuntao, ZhaoOsaki, MitsuhikoChuntova, Pavlina D.Oshima, TadayukiChurg, AndrewOsti, Mattia FalchettoChurov, AlexeyOstrowski, MarcinChutipongtanate, SomchaiOstróżka-Cieślik, AnetaCiardo, SilvanaOswald, FranzCiarimboli, GiulianoOta, AtsuhikoCiccarelli, RenataOta, TakayoCicchi, StefanoOthman, AhmedCiccolini, JosephOtowa, YasunoriCicènas, SauliusOtsuka, IsaoCicero, ArrigoOtt, GermanCichorek, MiroslawaOttaiano, AlessandroCidre-Aranaz, FlorenciaOttaviano, MargaretČierniková, SoňaOtterbein, LeoCieslar, GrzegorzOu, Da-LiangÇiftçi, Halil İbrahimOu, FangshuCiminale, VincenzoOuaissi, MehdiCimini, AnnamariaOudard, StephaneCimmino, AmeliaOuerfelli, OuathekCimmino, FloraOuled-Haddou, HakimCingolani, AntonellaOulhen, MarianneCinque, AlessandraOura, KyokoCinque, BenedettaOuseph, MadhuCioce, MarioOuzounis, ChristosCione, ErikaOve, RogerCipak Gasparovic, AnaOwczarczyk, KatarzynaCipak, LubosOwen, CarolynCipolletti, ManuelaOwen, GarethCiribilli, YariOwen, KatieCiriolo, MariaOwens, PhilipCiszewski, Wojciech MichałOyoshi, TakanoriCitro, SimonaOzaki, ShujiCittadini, GiuseppeOzeki, YasuhiroCitterio, DavideOzretić, PetarCives, MauroOzsvari, BelaCivita, ProsperoOzuner, GokhanClark, Dana FarengoPaakinaho, VilleClark, Geoffrey J.Paauwe, MadelonClark, John R.Pabst, AndreasClemons, NicholasPabst, ThomasClifford, Rachael E.Pacak, KarelClynes, DavidPach, RadoslawCocco, StefaniaPaciotti, MarcoCochonneau, DenisPackiam, Vignesh T.Cochrane, Dawn R.Padi, SathishCocozza, SergioPadler-Karavani, VeredCocuzza, SalvatorePadmanabhan, AchuthCocuzza, Salvatore GiuseppePadmanabhan, JayaCodacci-Pisanelli, GiovanniPagano, Allan F.Coffey, KellyPagano, NicoCogliati, BrunoPagella, FabioCognasse, FabricePagenstecher, AxelCohen, IdanPagliara, Monica MariaCohen, MariePagliuca, SimonaCohen-Gogo, SarahPagotto, SaraCokan, Kaja BlagotinšekPaholcsek, MelindaColaço, Bruno JorgePahwa, RomaColavito, Sierra A.Paíno, TeresaCole, LauraPainschab, MatthewCole, Trevor R. P.Paireder, MatthiasColecchia, MaurizioPal, AjayColell, AnnaPal, KrishnenduColetti, DarioPala, AndrejCollavin, LicioPalacios Calvo, JoseCollawn, Sherry S.Palacios García, DanielaCollins, Christopher M.Palaia, GaspareCollins, DenisPalamà, Ilaria Elena LenaCollura, AdaPalazzari, ElisaColnot, SabinePalazzo, ElisabettaColombo, MichelaPalazzo, LucaColomo, LluísPaleari, LauraColonna, GiovanniPaleologu, ConstantinColquhoun, Steven D.Palladini, AriannaConceição, NatérciaPallagi, PetraConciatori, FabianaPallavi, ManralConde-Estévez, DavidPallavicini, GianmarcoCondelli, ValentinaPalles, ClaireCondello, SalvatorePalleschi, AlessandroCondorelli, Daniele FilippoPalmer, Gregory M.Condorelli, GerolamaPalmieri, GiuseppeConigliaro, AlicePalmieri, Lola JadeConlon, NeilPalmieri, MariaConnolly, MichaelPalmqvist, RichardConoci, SabrinaPalomo, LauraConrad, David M.Palumbo, CarlottaConsalvi, ValerioPalumbo, Giuseppe A.Console, LaraPaluszczak, JaroslawConsonni, Francesca MariaPampena, RiccardoConstantinescu, IleanaPan, Chun-HsuConstantinidou, AnastasiaPan, EdwardConstanzo, JuliePan, JiayiContaldo, MariaPan, Wen-YuConti, AlfredoPanaro, FabrizioConti, PioPanda, AmareshCools-Lartigue, JonathanPandey, AmitCopeland, NikkiPandey, GauravCopland, MhairiPandey, RajeevCoppa, JorgelinaPandey, RituCoppes, RobPangault, CélineCoppey, MathieuPanigrahi, GatikrushnaCorbet, CyrilPanneerdoss, SubbarayaluCorbo, VincenzoPantalone, Mattia RusselCordani, NicolettaPantel, KlausCordenonsi, MichelangeloPantelyushin, StanislavCorey, SethPanzuto, FrancescoCormio, GennaroPaolillo, CarmelaCornelis, FrancoisPaolino, GiovanniCornelius, DenisePaoluzzi, LucaCorniola, Marco V.Paone, GaetanoCorominas, MontserratPapaccio, FedericaCorrenti, MargheritaPapachristodoulou, AlexandrosCorsaro, AlessandroPapachristos, Alexander J.Corsi, FabioPapadaki, CharaCorsi, LorenzoPapadaki, MariaCorso, SimonaPapadakos, KonstantinosCorti, ChiaraPapadimitriou, ChristosCorvalán, Alejandro H.Papadodima, OlgaCorvatta, LauraPapadopoulos, OthonCorvigno, SaraPapamatheakis, Joseph (Sifis)Costa, ClotildePapassotiriou, IannisCosta, José LuisPapoutsoglou, PanagiotisCosta, Maria Do CéuPappa, AglaiaCosta, MartaPaprocka, JustynaCostarelli, LeopoldoPaprottka, Philipp M.Costes, NicolasParadela, SabelaCote, Gregory M.Paranjape, AnuragCôté, HélèneParaskeva, EfrosyniCotoi, LauraParat, Marie-OdileCottini, FrancescaPardal-Refoyo, José LuisCouchman, JohnPardo-Pérez, MariaCoucke, WimParekh, ChintanCouttet, PhilippeParente, PaolaCouvineau, AlainParet, ClaudiaCovarelli, PieroParida, SheetalCoveñas, RafaelParis, FrancoisCoward, JermainePark, BoyoungCowden Dahl, KarenPark, Henry S.Cowley, Shaun M.Park, Hyun WooCox, DiannePark, Hyung SeokCoyle, BethPark, HyunjinCrabb, JohnPark, In JaCraig, Amanda J.Park, Jee SooCraig, MorganPark, Jeong A.Craig, Stephanie G.Park, Jong KookCramer, Gwendolyn M.Park, Ki-cheongCravo, MaríliaPark, Kyung ChanCrawford, Howard C.Park, Kyung SunCrawford, LisaPark, Mi KyungCrescioli, SilviaPark, Sue K.Creta, MassimilianoPark, Thomas In-HyeupCrezee, HansPark, WooramCrinò, Stefano FrancescoParker, SethCrisa, ElenaParkinson, E. KennethCrisafulli, GiovanniParmigiani, ElenaCristalli, GiovanniParolini, IsabellaCristina, MüllerParonetto, Maria PaolaCristofanilli, MassimoParra-Membríves, PabloCronauer, MarcusParrinello, GiampieroCronin, Patricia A.Parrozzani, RaffaeleCrooks, Daniel R.Parsons, JasonCros, JérômePartanen, MaritaCrosas, BernatParys, MaciejCroset, MartinePascale, RosaCrowe, DavidPascali, GiancarloCrucitta, StefaniaPascual, GloriaCrucitti, PierfilippoPasek, MałgorzataCrulhas, Bruno PereiraPasetto, AnnaCrum, ChristopherPasmant, EricCsapo, EditPasqualetti, GiuseppeCsizmadi, IlonaPasquali, ClaudioCsoka, MonikaPasquier, EddyCuadrado, MyriamPasseri, BenedettaCuatrecasas, MiriamPastorek, JaromirCubero, Francisco JavierPastoret, CédricCuccia, FrancescoPastorino, LorenzaCuenda, AnaPastré, DavidCui, FengPata, GiacomoCullen, JenniferPatankar, ManishCumbo, CosimoPatel, Girijesh KumarCumming, PaulPatel, NibeditaCuneo, AntonioPatelarou, AthinaCunningham, Jessica J.Pateras, Ioannis S.Cuocolo, RenatoPatócs, AttilaCurrie, MargaretPatrone, MauroCuschieri, KatePatrono, DamianoCusumano, DavidePatsoukis, Nikolaos E.Cvek, JakubPatterson, PandoraCvek, UrskaPattnaik, BikashCymbaluk-Ploska, AnetaPaudel, BishalCzarnecka, KarolinaPaudel, Keshav RajCzarniecka, AgnieszkaPaul, DennisCzarnomysy, RobertPaul, Manash K.Czyż, JarosławPaulides, MaartenD′Agnano, IgeaPaulo, AntónioD′agostino, DonnaPaulo, PaulaD′Agostino, MattiaPavlik, EdwardD′Agostino, Vito GiuseppePavlik, Edward J.D′Aguanno, SimonaPavlin, MojcaD′alessio, AlessioPavlova, ElenaD′Alò, FrancescoPavlova, GalinaD′Angelo, FrancescoPavone, Mary Ellen G.D′arcy, PádraigPaweł, KalińskiD′Errico, AntoniettaPawelczyk, TadeuszD′Onofrio, GiuseppePayabvash, Seyedmehdi Mehdi D.D′Onofrio, MirkoPayne , AnnetteD′Onofrio, NunziaPazgan-Simon, MonikaD′Orazi, ValerioPearl, Michael L.Da Silva, Luís PintoPecorari, GiancarloDa Vià, MatteoPecsi, DanielDabaja, BouthainaPedrosa, PedroDada, ReyadPei, YonggangDahal, LekhPeiris, CaseyDai, WeiPeitl, PetraDal Bo, MichelePeitzsch, ClaudiaDal Col, JessicaPeixoto, AndreiaDall, GenevievePejchal, JaroslavDalla Palma, Anna BenedettaPęksa, RafałDalla Vecchia, Laura AdelaidePellegrini, CristinaDamaghi, MehdiPellegrini, ManuelaDamascelli, BrunoPellegrino, MarshaDamato, BertilPellegrino, PaoloDambrauskas, ZilvinasPellegrino, RossellaDamiani, DanielaPellerino, AlessiaDamm, FrederikPelletier, JoffreyDammann, PhilippPellicano, RinaldoDammann, ReinhardPeltomäki, PäiviDaneshvar, Michael A.Pemov, AlexanderDanforth, David N.Peña-Chilet, MariaDang, Tuyen T.Peña-Chilet, MaríaDaniels, Mark J.Peña-Llopis, SamuelDanila, EdvardasPenalva, LuizDanilenko, MichaelPende, DanielaDanis, JuditPeng, DunfaDann, EldadPeng, I-ChenDanti, SerenaPenninckx, SébastienDanysh, Heather E.Pennock, NathanDaponte, AlexandrosPentimalli, FrancescaDarbin, OlivierPeperzak, VictorDario, Mandato VincenzoPera, AlejandraDariš, BarbaraPeralto, Jose Luis RodriguezDarling-Reed, Selina F.Peran, IvanaDarwiche, KaidPerego, MichelaDas, GokulPereira Lemos, JuliaDas, NupurPereira, DavidDas, PrasantaPereira, Patrícia A.Dasari, PallavePereira, Sofia S.Dasari, SubramanyamPereira-Veiga, ThaisDasgupta, ParamitaPérès, Elodie A.Dash, SabyasachiPeretti, SaraDash, SrikantaPérez Romasanta, Luis AlbertoDaskalakis, KosmasPerez, KimberlyDastoli, StefanoPérez-García, José ManuelDaubon, ThomasPerez-Hernandez, ConcepciónDavid Cordonnier, Marie HélènePerez-Ruiz, ElizabethDavid, MutchPérez-Santos, MartínDavidson, Brian R.Perez-Stable, CarlosDavie, JudithPergolizzi, DeniseDawnay, AnnePerini, GiordanoDawson, Michelle R.Peris, KettyDay, Andrew S.Perła-Kaján, JoannaDay, Chi-pingPerna, GiuseppeDay, Regina M.Pérol, DavidDayyani, FarshidPeron, JulienDe Araújo Júnior, Raimundo FernandesPeroni, EdoardoDe Assis, Leonardo V. M.Perra, TeresaDe Baene, WouterPerrakis, AristotelisDe Bari, LidiaPerrone, Anna MyriamDe Barrios, OriolPerrone, FrancescoDe Bree, EelcoPersano, StefanoDe Bree, RemcoPersichetti, PaoloDe Carvalho, Litia AlvesPersico, MarcelloDe Clercq, EvaPersson, EmmaDe Cristofaro, TizianaPersson, JennyDe Felice, FrancescaPeters, ChristianDe Gregorio, VincenzaPetersen, Ole WilliamDe Haas, ValeriePethő, ZoltánDe Jaeghere, Emiel A.Petitprez, FlorentDe Jong, Daphne D.Petrella, FrancescoDe Jong, Florus C.Petrik, JimDe Jong, Igle JanPetrone, Maria ChiaraDe Jong, MoniquePetroni, MarialauraDe Juan Romero, CaminoPetros, John A.De Klein, AnneliesPetrov, AntonDe La Parra, ColumbaPetrova, BoryanaDe La Parte, Borja HerreroPetruczynik, AnnaDe La Peña, Francisco AyalaPetry, AndreasDe La Roche, MarcPettinato, GiuseppeDe La Rosa, JorgePettit, KristenDe La Rubia, JavierPeulen, OlivierDe Ligt, KellyPeyrl, AndreasDe Los Toyos, Juan R.Pezone, AntonioDe Luca, AnastasiaPezzilli, RaffaeleDe Marchi, ElenaPezzolo, AnnalisaDe Marco, FedericoPfahlberg, AnnetteDe Maria, RaffaellaPfahlberg, Annette B.De Martino, Maria CristinaPfister, Astrid S.De Matos Simoes, RicardoPhatarpekar, PrasadDe Mattia, ElenaPhelan, James J.De Melo Porcari, AndréiaPhélip, Jean MarcDe Melo-Diogo, DuartePhilipp, ManfredDe Mestier, LouisPhillips, Kelly A.De Meyts, PierrePiana, MicheleDe Miglio, Maria RosariaPicardo, Sarah L.De Miguel-Pérez, DiegoPiccardo, ArnoldoDe Nigris, FilomenaPiccin, AndreaDe Nonneville, AlexandrePiccinin, ElenaDe Nully Brown, PeterPicco, VincentDe Paoli, AntoninoPicimbon, Jean-FrançoisDe Petro, GiuseppinaPiechota-Polanczyk, AleksandraDe Pinho, Paula GuedesPierelli, LucaDe Reijke, Theo M.Pierfrancesco Francesco, BassiDe Reuver, PhilipPieroni, LuisaDe Ridder, MischaPietra, GabriellaDe Robertis, MariangelaPietrangelo, LauraDe Rossi, AnitaPietrusiński, MichalDe Santis, Maria ChiaraPietruszewska, WiolettaDe Santo, CarmelaPigazzi, MartinaDe Sarkar, NavonilPignot, GéraldineDe Sepulveda, PauloPijnenborg, HannyDe Sousa-Coelho, Ana LuísaPilarsky, ChristianDe Terlizzi, FrancescaPilatus, UlrichDe Totero, DanielaPileri, StefanoDe Velasco, GuillermoPilleron, SophieDe Velasco, Marco A.Pillozzi, SerenaDe Vicente, Juan CarlosPilmane, MāraDe Vincenzo, RosaPina, AnnickDe Vita, AlessandroPina, CristinaDe Wilde, BramPincus, SethDe, TanimaPindyurin, Alexey V.Deaglio, SilviaPineda, SilviaDealwis, Chris G.Piñeiro, RobertoDean, MichaelPink, DanielDeandreis, DésiréePinkawa, MichaelDeantonio, LetiziaPintea, BogdanDeb, SubrataPinto, CarmineDecarolis, BorisPinto, EleonoraDeckert, MarcelPinto, FilipeDe-Colle, ChiaraPinto, MafaldaDecottignies, AnabellePinzani, PamelaDeevska, GerganaPioli, ClaudioDefilippi, PaolaPiotrowska, EwaDekanic, AndreaPiovesan, AlessandroDel Bianco, PaolaPiperi, ChristinaDel Favero, GiorgiaPiperno-Neumann, SophieDel Giudice, FrancescoPipkorn, PatrikDel Mastro, L.Piri, RezaDel Mistro, AnnarosaPirich, ChristianDel Rivero, JaydiraPiris Pinilla, Miguel ÁngelDel Rosso, MarioPirtea, LaurentiuDel Sal, GianninoPisani, CarlaDel Vecchio, SilvanaPisani, Laura FrancescaDel Zingaro, MichelePiva, RobertoDel Zotto, GennyPiva, TerrenceDelhomme, Tiffany M.Pizzi, MarcoDelie, FlorencePlacer, CarlosDell′Amore, AndreaPlanque, ChrisDell′Aversana Orabona, GiovanniPlantinga, MaudDella Bella, ChiaraPlatta, HaraldDella Pepa, Giuseppe MariaPlattner, RinaDella Valle, SerenaPlazzer, John PaulDelle Cave, DonatellaPlesa, AdrianaDellis, OlivierPluck, GrahamDello Sbarba, PersioPluquet, OlivierDemehri, ShawnPlymate, Stephen R.Demetter, PieterPocard, MarcDemicheli, RomanoPodar, KlausDe-Miguel, ManuelPoder, Thomas G.Demirci, HakanPodhorska-Okołów, MarzenaDemirkhanyan, Lusine H.Podlipnik, SebastianDeng, QuPoggio, FrancescaDeng, YoupingPohlan, JulianDengel, AndreasPoirier, DonaldDenis, JeromePoirier, Guy G.Denisov, EvgenyPoirot, MarcDenizot, YvesPolakowski, Nicholas J.Dennany, LynnPolańczyk, AndrzejDennis, Leslie K.Polano, MaurizioDenys, AlbanPolap, DawidDeore, Prashant S.Polese, LinoDepau, LorenzoPolesel, JerryDepciuch, JoannaPolesello, CédricDeptuła, MilenaPolgarova, KamilaDerks, J. L.Poli, ValeriaDerksen, Patrick W. B.Polici, MichelaDerlin, ThorstenPolimeno, LorenzoDerwich, KatarzynaPoliseno, LauraDesantis, VanessaPolishchuk, RomanDesaphy, Jean-FrançoisPollice, AlessandraDesouki, Mohamed M.Pollok, Karen E.Deutsch, AlexanderPolz-Dacewicz, MalgorzataDevaux, BertrandPompano, RebeccaDevillier, RaynierPond, Amber L.Devine, KatiePond, GregoryDey, Debasish KumarPongracz, Judit E.Dey, KamolPonik, SuzanneDey, NandiniPonikwicka-Tyszko, DonataDey, PriyankaPons, SebastianDey, ShuvashisPonz-Sarvise, MarianoDhimolea, EugenPop, LaurentiuDhungel, BijayPopanda, OdiliaDi Agostino, SilviaPoplawski, PiotrDi Cataldo, AndreaPopli, PoojaDi Donato, MarziaPopovic, SnezanaDi Franco, SimonePopper, Helmut H.Di Giammartino, Dafne CampigliPoptani, HarishDi Grezia, GraziellaPorcu, EleonoraDi Mario, FrancescoPorcu, LucaDi Nunno, VincenzoPorpodis, KonstantinosDi Palma, TinaPortella, GiuseppeDi Paola, ValerioPortman, NeilDi Pietro, PietroPorto, BeatrizDi Renzo, LiviaPoschke, IsabelDi Renzo, Maria FlaviaPoškus, EligijusDi Rorà, Andrea GhelliPossamai, Lucia A.Di Saverio, SalomonePosthaus, HorstDi Sipio, TraceyPotempa, Lawrence A.Di Spiezio Sardo, AttilioPotez, MarineDi Tommaso, LucaPotočnik, NejkaDi Trapani, EttorePouget, Jean-PierreDiacono, DomenicoPouliakis, AbrahamDianzani, UmbertoPouliquen, Daniel LoïcDiaz, AbbeyPourbaghi, MiladDiaz, FernandoPourkarim, Mahmoud RezaDiaz-Flores, LucioPoxleitner, Philipp J.Díaz-García, DianaPoyet, Jean-LucDíaz-Rodríguez, ElenaPozzi, NicolaDidier, ChristinePrabhu, Antony HeroldDidilescu, AndreeaPrabhu, LakshmiDiefenbach, Russell J.Prada, IlariaDiepstra, ArjanPranckevičienė, AistėDierge, EmelinePrante, OlafDieterich, LotharPrasad, VikasDietrich, CharlesPrasanna, PrateekDijk, FrederikePravettoni, GabriellaDillman, Robert O.Prawitt, DirkDimanche-Boitrel, Marie-ThérèsePreca, Bogdan TiberiusDimitrakopoulos, Foteinos-IoannisPreda, LorenzoDimitriadis, Emilios K.Prehn, JochenDimri, ManaliPrehn, Jochen H. M.Dinalankara, WikumPreti, MarioDindelegan, George CalinPreudhomme, ClaudeDing, Han-FeiPrevarskaya, NataliaDing, KeyuePrevis, Rebecca A.Ding, LingwenPrezioso, LuciaDinh, Claire T.Prigerson, Holly G.Dini, ValentinaPrimignani, MassimoDinic, JelenaPrince, Mark E.Dinis-Oliveira, Ricardo JorgePrise, Kevin M.Dionyssiou, DimitriosPritchard, CatrinDiorio, CarolinePrivat-Maldonado, AngelaDirven, LindaPrivette Vinnedge, LisaDisciglio, VittoriaProescholdt, MartinDittmer, JurgenProvencio, MarianoDixon, Stephanie B.Pryczynicz, AnnaDjerada, ZoubirPrzemek, KrawczykDmitriev, RuslanPsoroulas, SerenaDmitrieva, Renata I.Psyrri, AmandaDoberstein, KaiPudakalakatti, Shivanand M.Dobos, GaborPugliese, MariagabriellaDobrescu, AmadeusPuhka, MaijaDobruch, JakubPuig-Butillé, Joan AntonDobruch-Sobczak, KatarzynaPuijk, RobbertDobrzyn, PawelPuisset, FlorentDoghman, MabroukaPula, BartoszDoglio, AlainPuladi, BehrusDogra, SamritaPuliani, GiuliaDoherty, MarkPulido, RafaelDolivo, DavidPulito, ClaudioDomagala-Kulawik, JoannaPulliero, AlessandraDomanski, HenrykPusch, StefanDomenech, MaribellaPuustinen, PietriDomenico, MascagniPuy, CristinaDomingo, EnricPylayeva-Gupta, YuliyaDomínguez-Valentín, MevPyronnet, StéphaneDommering, Charlotte J.Qaddoumi, IbrahimDomoto, TakahiroQdaisat, AihamDonahoe, LauraQi, XiaoyangDonati, ChiaraQi, Yanfei (Jacob)Dong, LiangQi, YueDong, LianhuaQian, YanrongDongryull, OhQiao, FangfangDonner, DavideQin, WeijunDoretto, PaoloQin, ZhiqiangDoria, CataldoQing, TaoDorleijn, Desirée M. J.Qiu, BinDorling, LeilaQiu, GuangyuDormond, OlivierQiu, Jiantai TimothyDorsman, JosephineQiu, ZhenDos Anjos Pires, MariaQu, NingDotan, ZoharQu, PengDraghi, AriannaQuadri, MarikaDragoni, SilviaQuadri, Syed A.Drake, Penelope M.Quagliariello, VincenzoDrakos, EliasQuaia, EmilioDreassi, ElenaQuante, MichaelDreyer, Tobias F.Quarta, AlessandraDrid, PatrikQuatrini, LindaDriessen, ChristophQuattro, Joseph M.Dröge, Leif HendrikQuax, PaulDrott, JennyQuax, Wim J.Droz, JeanpierreQue, JennyDrozd-Sokołowska, JoannaQuek, CameliaDryahina, KseniyaQuiding-Järbrink, MarianneDu, JuanQuinn, GwendolynDu, WentingQuinn, Gwendolyn P.Du, XuexiangR. Naik, AkshataDuan, JialeiRaber-Durlacher, Judith E.Duarte, AntonioRabien, AnjaDubash, Taronish DorabRada, MiranDubois, Claire M.Radeghieri, AnnalisaDubois, LudwigRades, DirkDubrovska, AnnaRadisavljevic, ZivDuc, Nguyen MinhRadochová, VladimíraDuca, LaurentRadosavljević, TatjanaDuca, LorenaRadosevic-Robin, NinaDuchi, SerenaRadu, BeatriceDucret, ThomasRadulovic, MarkoDuensing, StefanRadziejewska, IwonaDufresne, ArmelleRadzki, DominikDuggan, ShaneRafael, DianaDugué, Pierre-AntoineRafaelsen, Søren RafaelDuhamel, MarieRaffone, AntonioDulskas, AudriusRaghavendra, AksharaDumaz, NicolasRagno, PiaDumic, JerkaRagoussis, JiannisDumitrascu, TraianRaguse, Jan-DirkDummer, ReinhardRahimi, SiavashDumont, FrédéricRahman, ArmanDumont, Sarah NoémieRahman, NafisDumoulin, Franz LudwigRahmani, RediDunlap, NealRai, GaneshDunlop, ElaineRai, KhushminderDuque-Afonso, JesusRai, VikrantDurán-Poveda, ManuelRaia, MaddalenaDuranti, ClaudiaRaikhy, GauravDuranti, SimonaRaimondo, DiegoDurdik, MatusRaja, Iruthayapandi SelestinDurgo, KsenijaRajagopalan, AnugrahaDurlik, MagdalenaRajasekaran, NarendiranDurocher, FrancineRajendran, PraveenDurrieu, FrancoiseRaji, IdrisDuskey, Jason ThomasRajkumar Singh, KalraDuso, BrunoRajpurohit, SurendraDuś-Szachniewicz, KamilaRakislova, NataliaDutil, JulieRamakrishnan, VijayDutta, ArijitRamasamy, MohankandhasamyDutta, PranabanandaRamazanov, BulatDutta, SamikshanRambaldi, AlessandroDüzgüneş, NejatRameshwar, PranelaDvorankova, BarboraRamírez, Cristina M.Dwarkasing, Roy S.Ramírez-Labrada, ArielDye, Danielle E.Ramirez-Peña, EsmeraldaDymock, Brian WilliamRamon Torrell, Josep MariaDymova, MayaRamos, EmilioDziegiel, PiotrRamos-Garcia, PabloDziegielewska, BarbaraRampazzo, ElenaDzięgielewska-Gęsiak, SylwiaRampias, TheodorosDzikiewicz-Krawczyk, AgnieszkaRamsköld, DanielEast, Michael P.Randazzo, AntonioEastman, AlisonRanganna, KasturiEatrides, Jennifer M.Rangel-Pozzo, AlineEaves-Pyles, TonyiaRani Banik, GouriEberhart, CharlesRanieri, DaniloEble, JohannesRanieri, GirolamoEbstein, FrédéricRanjit, SabinaEccher, AlbinoRansac, StéphaneEchchannaoui, HakimRantala, Juha K.Echevarría, EnriqueRao, GeetaEckert, AlexanderRao, MahadevEckert, Alexander W.Rao, RajiniEckrich, JonasRao, Tata NageswaraEckschlager, TomasRaoul, WilliamEdderkaoui, MouadRapi, StefanoEdeline, JulienRapic, SaraEden, AmirRapino, CinziaEdfors, FredrikRapisarda, VenerandoEdwards, ClaireRasche, LeoEeltink, Corien M.Raschke, FelixEersels, Jos L.Raška, MilanEfebera, Yvonne A.Rasmussen, Torben RiisEferl, RobertRasouly, Hila MiloEfremov, DimitarRaspagliesi, FrancescoEgashira, NobuakiRasul, SazanEgger, JanRatajska, MagdaEhata, ShogoRather, GulamEhrlich, MelanieRathert, PhilippEichbaum, MichaelRatti, StefanoEichenauer, Dennis A.Rattray, ZahraEichhorn, FlorianRaundhal, MaheshEichhorn, PieterRaup-Konsavage, WesleyEichler, MartinRausei, StefanoEisendle, KlausRauso, RaffaeleEisenstat, David D.Rav Acha, MosheEisermann, KurtisRava, MartaEiz-Vesper, BrittaRavaioli, MatteoEkert, Paul G.Ravanelli, MarcoEkkernkamp, AxelRawat, ManmeetEksi, Sebnem EceRawicz-Pruszyński, KarolEksioglu, Erika A.Ray, ArghyaEl Sharouni, Mary-AnnRay, UpasanaElander, NilsRayar, MichelEldfors, SamuliRazzokov, JamoliddinElfer, KatherineRazzuoli, ElisabettaEl-Gewely, Mohamed RaafatRbah-Vidal, LatifaEliçin, OlgunRead, MatthewEliseeva, IrinaReader, JocelynElkabets, MosheReagan, John L.Elkrief, ArielleReali, EvaEllinger, JörgReddick, Wilburn E.El-Mesery, MohamedReddy Bonam, SrinivasaEloranta, SandraReddy, AswinEl-Rifai, WaelReddy, KesavaElsawa, Sherine F.Reddy, Sudhir PuttyElsayad, KhaledRedina, OlgaElsherbini, Ahmed M.Redini, FrancoiseElsherbiny, MarwaRedondo Muñoz, JavierEmfietzoglou, DimitrisReed, Amy McCartEmons, GünterReese, Jennifer BarskyEmran, Abdullah AlReeves, Helen L.Emri, GabriellaRegel, IvonneEndo, MakotoReggiani, FrancescaEngels, YvonneReginato, MauricioEnnour-Idrissi, KaoutarRegnery, SebastianEnokida, TomohiroRehfeld, Jens FrederikEnomoto, MasaruReich, AdamEnose-Akahata, YoshimiReich, WaldemarEnrico, GringeriReig, ÒscarEntz-Werle, NatachaReimer, Daniel U.Eoli, MaricaReinders, JoergEr, Tze-KiongReindl, KatieEravuchira, Pinkie J.Reiner, Anne S.Erben, PhilippReinhardt, HeikeErdmann, Joris I.Reis, Rui ManuelEri, S. SrivatsanReis, Sara S.Eric, EspinosaRen, XiangEricson, Marica B.Rengarajan, VenkatakrishnanEricson, Marna E.Renzo, VannaEriksson Steigen, SonjaResheq, Yazid J.Erneux, ChristopheResnick, IgorErnst, CorinnaRestaino, StefanoErokhin, MaksimRetsky, MichaelErovic, Boban M.Rettig, EleniEscargueil, AlexandreRettinger, EvaEscolà-Gil, Joan CarlesReu, Frederic J.Escorcia, Freddy E.Revelli, LucaEscors, DavidRevuri, VishnuEscudier, Jean-MarcReyes-Aldasoro, Constantino CarlosEso, YujiReymond, MarcEspel, EnricReyners, Anna K. L.Esposito, Maria TeresaRho, Jin KyungEsposito, Maria ValeriaRialland, MickaëlEsposito, RobertoRibatti, DomenicoEstanyol, Josep MariaRibero, SimoneEsteves Da Silva, Joaquim C. G.Ricardo, SaraEstévez, Laura GarcíaRicchetti, FrancescoEstrada, Marta F.Ricci, AlbertoEu, PeterRicci, Angela DaliaEufemi, MargheritaRicci, FrancescaÉva, MikóRicci, GiovannaEvans, D. GarethRicci, GiuliaEvans, Gareth R.Ricciardi, Maria RosariaEvanson, Nathan K.Rich, Thomas C.Evert, MatthiasRichardson, AlanEvgin, LauraRichardson, Sandra R.Evrard, Solène M.Riches, John C.Ewald, CollinRichmon, JeremyEykyn, ThomasRichter, Günther H. S.Ezzat, ShereenRichter, SusanEzzidin, SamerRicke, WilliamFabbri, FrancescoRider, PaulFabbri, MullerRidola, LorenzoFabbrizi, Maria RitaRidolfi, LauraFaber, Anthony C.Riefolo, MattiaFabi, AlessandraRiegman, Peter H. J.Fabiani, RobertoRieken, StefanFabris, LucaRiether, CarstenFabrizio, MontecuccoRigacci, LuigiFacchiano, AntonioRigalli, Juan PabloFacciorusso, AntonioRigaud, MichelFacoetti, AngelicaRigby, Matthew H.Fadda, GuidoRigopoulou, EiriniFadhel, Muhannad N.Riis, Margit L. H.Faggiano, AntongiulioRim, Chai HongFagioli, FrancaRimondini, LiaFagoonee, SharmilaRingdén, OlleFahraeus, RobinRiond, JoëlleFaini, Angelo CorsoRiou, OlivierFaísca, PedroRiquelme, ArnoldoFalahat, RanaRishi, ArunFalcioni, RitaRiva, GiuliaFalcone, ItaliaRiva, GiuseppeFalk, StephenRivero, FranciscoFalzone, LucaRizcallah, EdmondFan, MeiyunRižner, Tea LanišnikFanciulli, GiuseppeRizzo, AlessandroFandrey, JoachimRizzo, AngelaFanetti, GiuseppeRizzolio, FlavioFang, QingmingRoberg, KarinFang, Wen-LiangRoberts, David D.Fanoni, DanieleRoberts, JanetFanti, AlessandroRoberts, TaraFanti, StefanoRobichaux, JacqulyneFantini, SebastianRobinson, JamesFarace, FrançoiseRobitaille, MélanieFarah, Camile S.Roccaro, AldoFardilha, MargaridaRocco, GaetanoFaria-Ramos, IsabelRochani, AnkitFarnetani, FrancescaRoche, SergeFarolfi, AlbertoRodeberg, DavidFarooqi, Ammad AhmadRoderburg, ChristophFarquhar-Smith, W. PaulRodland, Karin D.Farruggia, PieroRodolfo, MonicaFarrugia, Mark K.Rodrigo, Juan P.Fasano, MorenaRodrigues Santiago, LeandroFato, RomanaRodrigues, DarioFattizzo, BrunoRodrigues, Pedro MiguelFaucz, Fabio R.Rodríguez, CristinaFaviana, PinucciaRodriguez, F. DavidFaymonville, Marie-ElisabethRodríguez, JavierFazzari, JenniferRodríguez-García, AlbaFeda, H. HamdanRodriguez-Lafrasse, ClaireFedele, MonicaRodriguez-Nogales, CarlosFedeli, AlessandroRodríguez-Santamarta, TaníaFehrenbach, UliRodriguez-Vargas, Jose ManuelFei, MiaoRoedel, FranzFeier, Diana SorinaRoels, JulietteFelicetti, FedericaRoesch, AlexanderFelidj, NordinRoessner, EricFeliu, JaimeRofi, EleonoraFélix, AnaRogakou, EmmyFelli, Maria PiaRogalska, AnetaFelsenstein, MatthäusRogatto, SilviaFeng, HuiRogowski, PaulFeng, WanjuanRoh, Tae HoonFenton, TimRohde, StefanFenyö, DavidRöhrlich, Pierre SimonFeo, ClaudioRojiani, Mumtaz V.Feo, FrancescoRojo, Jose M.Feraco, PaolaRok, JakubFerdek, PawełRokavec, MatjazFerdinandus, JustinRola, RadosławFerioli, MartinaRoll, WolfgangFermi, MatteoRomagnoli, StefanoFernandes, ElisabeteRoman, MaciejFernandes, GabrielaRomani, AndreaFernandes, RicardoRomanini, AntonellaFernández Cabada, TamaraRomano, AlbertoFernández Casanova, LucíaRomanov, VictorFernández De Larrea, CarlosRoma-Rodrigues, CatarinaFernandez, Harvey R.Rombouts, KristaFernandez-Botran, RafaelRomee, RizwanFernández-Medarde, AlbertoRomero Collado, AngelFernández-Sáiz, VanesaRomero-Masters, JamesFernández-Tomé, María Del CarmenRomeyke, TobiasFerrara, BenedettaRomiti, Giulio FrancescoFerraresi, AlessandraRommelaere, JeanFerrari, DarisRompianesi, GianlucaFerrari, MarcoRóna, GergelyFerreira, CarolinaRonchetti, DomenicaFerreira, FernandoRonellenfitsch, UlrichFerreira, RodrigoRong, Chan KuanFerrero, GiulioRong, RuichenFerri, FlaminiaRonot, MaximeFerriere, FrançoisRoodhart, JeanineFerroni, PatriziaRoper, NitinFerrucci, MichelaRoque, NataliaFerrucci, Pier FrancescoRos, JavierFeuillard, JeanRos, SusanaFigueiredo, Carlos R.Rosa-Caldwell, Megan E.Figueroa, AngélicaRosado, JuanFilbet, MarilèneRosahl, SteffenFiligheddu, NicolettaRosanò, LauraFilipovic, MiodragRoschke, AnnaFilippi, LucaRose, MichaelFilippi, SilviaRoseline, GodboutFilippou, Panagiota S.Rosen, TheodoreFilippova, NataliaRosenbaum, ThorstenFina, EmanuelaRosenberg, Jens T .Finazzi, TobiasRoshan, AmitFinke, DanielRosol, ThomasFinkielstein, CarlaRoss, Jeffrey S.Fionda, BrunoRossi, FrancescaFionda, CinziaRossi, MaijaFiore, DaniloRossi, PaoloFiorelli, AlfonsoRossi, Roberta ElisaFiorentini, CarlaRossiello, FrancescaFiorentini, GiammariaRossini, DanieleFiori, EnricoRosswog, CarolinaFiorica, FrancescoRossy, JérémieFirczuk, MalgorzataRotblat, BarakFischer, MartinRothberg, PaulFischer, Nicholas W.Rotte, AnandFischer, NicoleRoulois, DavidFischer-Fodor, EvaRoumeguère, ThierryFitch, MargaretRousseau, AudreyFitzgerald, DavidRousselle, PatriciaFiz, FrancescoRovida, ElisabettaFlavin, RichardRovirosa, ÁngelesFleitas, TaniaRovo, AliciaFliedner, StephanieRoy, Soumyajit D.Flores-Montero, JuanRoyer-Pokora, BrigitteFlórez, ÁngelesRoyse, Kathryn E.Flouriot, GillesRozman, AlešFolini, MarcoRual, Jean-FrancoisFollo, Matilde YungRubben, AlbertFon Tacer, KlementinaRubio Retama, JorgeFonseca-Alves, Carlos EduardoRubis, BlazejFontana, RosaRubnitz, Jeffrey E.Fontanini, GabriellaRubtsova, Maria P.Font-Burgada, JoanRuchała, MarekFontenay, MichaelaRudd, Sean GFoote, Janet A.Rudelius, MartinaForay, NicolasRudolf, RuedigerForciniti, StefaniaRueda, BoFord, John C.Ruehl, HeikoForero-Castro, MaribelRuggeri, Rosaria M.Forest, FabienRuggiero, Maria RosariaFormanek, MartinRuhaak, Lucia ReneeFornasaro, StefanoRuiz, FacundoForsare, CarinaRuiz, Irene RiusForsberg, MatthewRumpold, HolgerFörsch, SebastianRund, DeborahFörschler, AnnetteRuotsalainen, JanneForschner, AndreaRusak, MalgorzataForster, MichaelRusanova, IrynaFort, PhilippeRussell, NicolaForte, Iris MariaRussell, StepehenFortenberry, YolandaRusso, AlessandroFoschini, M. P.Russo, AngelaFossati, PieroRusso, AntonioFox, Joanna L.Russo, DanielaFracasso, GiulioRusso, GiorgioFradin, DelphineRusso, GiovannaFragomeni, Simona MariaRusso, IsabellaFraile-Bethencourt, EugeniaRuszkowska-Ciastek, BarbaraFraizer, GailRutkowsk, PiotrFrampton, Adam E.Ruvo, MenottiFranceschini, GianlucaRuzgys, PauliusFrancesco, AnielloRuzov, AlexeyFrancescone, RalphRuzzene, MariaFranchet, CamilleRuzzenente, AndreaFranchi, MatteoRyan, AideenFrancia, SofiaRydén, LisaFrancini, EdoardoRyu (Yoo), Kyung HyunFrancis, HeatherRyu, BomiFrancis, Jasmine H.Ryu, Jung SuFrancisci, SilviaRyu, Keun HoFranck, JulienRzepakowska, AnnaFranco, LuisRzymowska, JolantaFranco, PierfrancescoSaar, MatthiasFrancolini, GiulioSabaawy, Hatem E.Francone, ElisaSabarimurugan, ShanthiFrancuz, TomaszSabatier, LaureFranko, JanSabatier, RenaudFranses, Joseph W.Sabatino, GiovanniFranz, LeonardoSabloff, MitchellFranzini, AncaSabol, MajaFraser, MichaelSacchini, VirgilioFrassanito, Maria AntoniaSaccilotto, RamonFrati, AlessandroSaccone, SalvatoreFratila, RalucaSaceda, MiguelFratino, LuciaSack, UlrichFrauenknecht, KatrinSaddik, BasemaFrazzi, RaffaeleSadhukhan, PritamFredolini, ClaudiaSadler, AnthonyFrega, GiorgioSaeki, IsseiFreitas, DanielaSafa, Ahmad R.Freitas, Lucas F.Safi, SeyerFremd, CarloSafránek, DavidFrese, KristopherSaglio, FrancescoFrew, Ian J.Sagnou, MarinaFrey, BenjaminSaha, BiswarupFreyer, David R.Saha, NirmalyaFrezzato, FedericaSaha, ShekharFribley, AndrewSahara, HiroekiFridrichova, IvanaSahgal, PranshuFrieboes, Hermann B.Sahu, Sanjaya KumarFriedl, Anna A.Saillard, CharlieFriedmann Angeli, José PedroSaini, SharanjotFriedrich, Christoph M.Saito, RyokoFriedrich, MircoSaito, RyutaFriedrichs, NicolausSaito, TsuyoshiFriesland, SigneSaito, YuichiFriess, HelmutSaitoh, TakayukiFrietze, SethSajesh, Babu V.Frigo, Daniel E.Sajjadi, ElhamFrolova, LiliyaSakai, KazukoFuchs, BrunoSakakura, NoriakiFuchs, Hans FriedrichSakamoto, ShinichiFuchs, HermanSakamoto, YasunariFuchs, OtaSakamoto, YuzuruFueger, Barbara J.Sakamuro, DaitokuFujihara, HisakoSakata, KiyohikoFujii, Shin-ichiroSakuma, KunihiroFujii, ShoichiSakwe, AmosFujii, TomomiSalagierski, MaciejFujii, YouichiSalar, AntonioFujimori, KoSalas, Lucas A.Fujimura, AtsushiSałat, KingaFujishima, FumiyoshiSalazar, IvanFujita, KazutoshiSaleh, Fatima A.Fujita, NaokiSaleh, MazenFujiwara, MasayukiSalehzadeh-Yazdi, AliFujiwara, NaotoSalgar, ShashikumarFujiwara, YukioSalhi, YahiaFukuoka, HidenoriSalido, MartaFukushima, HiroshiSalimi, MarziehFulci, ValerioSalla, MohamedFulton, LawrenceSalle, ValéryFumagalli Romario, UbertoSalomé Pires, AnaFumoto, ShintaroSalomon, CarlosFunahashi, AkiraSalto-Tellez, ManuelFunai, KazuhitoSalvatore, LisaFurlanetto, JennySalvatori, PietroFurler, Robert L.Samaja, MicheleFurqan Qadri, SyedSamalin, EmmanuelleFurtner-Srajer, JuliaSamaniego, RafaelFurukawa, TatsuhikoSamarzija, IvanaFurukawa, ToruSamasca, GabrielFusco, NicolaSambri, AndreaFushida, SachioSamec, MarekFütterer, JurgenSammarco, GiuseppeFutyma, KonradSamnick, SamuelGabbia, DanielaSamuels, BrianGabrić, DraganaSamykutty, AbhilashGabriel, EmmanuelSanawar, RahulGabrielli, BrianSánchez-Beato, MargaritaGabrielsson, SusanneSánchez-Bueno, FranciscoGabryel, PiotrSanchez-Elsner, TilmanGabryś, DorotaSánchez-Pérez, IsabelGaetano, CarloSancisi, ValentinaGagat, MaciejSanda, MiloslavGagner, Jean-PierreSandfeld-Paulsen, BirgitteGagos, SarantisSandhu, Shahneen K.Gaidano, GianlucaSanduleanu, SilviaGaidano, ValentinaSanguedolce, FrancescaGaidzik, VerenaSanità, GennaroGaikwad, HanmantSano, MakotoGainullin, MuratSansom-Daly, UrsulaGaipl, Udo S.Sansone, AnnaGaiser, TimoSansone, FrancescoGajek, ArkadiuszSantamaria-Martínez, AlbertGajewska, A.Santarelli, AndreaGajos-Michniewicz, AnnaSantini, DonatellaGalandrini, RicciardaSantonico, ElenaGalardy, Paul J.Santos, Joana M. O.Galbiati, SilviaSantos, MafaldaGaletta, DomenicoSantos, MirentxuGalibert, Marie-DominiqueSantos-Lozano, AlejandroGalindo, ReneSantos-Rocha, Rita AlexandraGallardo, FernandoSantpere, GabrielGallego, Óscar S.Santulli, GaetanoGalli, GiuliaSanz, Miguel A.Gallivanone, FrancescaSapalidis, KonstantinosGallo, GaetanoSaponaro, ConcettaGallo, JuanSarabia-Cobo, Carmen MaríaGallo, OresteSarantis, PanagiotisGallo, SalvatoreSardella, EloisaGallotta, ValerioSargi, ZoukaaGalvano, AntonioSarhadi, Virinder KaurGaman, Mihnea-AlexandruSarhan, DhifafGambacorta, Maria AntoniettaSarkadi, BalazsGambacorti-Passerini, CarloSarkar, AnujitGambardella, GennaroSarkar, SibajiGamberi, TaniaSarkozy, ClémentineGameiro, SteveSarma, PranjalGameiro, Steven F.Sarmento-Ribeiro, Ana BelaGamradt, PiaSarna, MichalGanau, MarioSarno, FedericaGangemi, RosariaSarobe, PabloGangopadhyay, SoumyashreeSarosiek, Shayna R.Ganguly, AnutoshSarrette, Jean PhilippeGani, Jonathon S.Sarria, Gustavo R.Ganju, AdityaSartor, Maureen A.Ganser, ArnoldSasada, TetsuroGao, AllenSasai, NoriakiGao, JingjingSasaki, MakotoGao, ShuaiSasaki, MotokoGaponenko, VadimSasaki, YasushiGarattini, EnricoSascha, RahnGaravaglia, SilviaSasso, Ferdinando CarloGarayoa, MercedesSastre-Serra, JorgeGarbossa, DiegoSathiyaraj, SrinivasanGarcía Gutiérrez, José ValentínSato, FuyukiGarcia Gutierrez, LuciaSato, HirokiGarcia Hernandez, Juan LuisSato, MarikoGarcía, Juán FernandoSato, ShuichiGarcia-Borron, Jose CarlosSato, YukiyasuGarcía-Escudero, RamónSato, YusukeGarcía-Flórez, LuisSatoh, TarohGarcia-Giralt, NataliaSatoi, SoheiGarcía-Martínez, AraceliSaulnier, OlivierGarcía-Otín, Ángel LuisSaulnier, RonGarcia-Rico, EduardoSauro, KharaGarcía-Rojo, MarcialSauter, Edward R.García-Silva, SusanaSavic, Lynn JeanetteGardner, Thomas A.Savic-Radojevic, AnaGarella, RacheleSavvidou, IoannaGarg, ManojSawabata, NoriyoshiGarí, EloiSawada, KojiGariglio, MarisaSawyer, Elinor J.Garje, RohanSayed, Ibrahim M.Garofalo, MariangelaScaife, Courtney L.Garrastachu, PuyScala, StefaniaGarrè, Maria LuisaScalabrin, MattiaGarrick, DavidScalco, ElisaGarrigue, PhilippeScarfò, LydiaGartel, AndreiScarpa, EdoardoGartner, FátimaScarpi, EmanuelaGasparotto, DanielaScatena, RobertoGasparri, RobertoSchache, AndrewGasparski, AlexanderSchäfer, GeorgiaGašperov, Nina MilutinSchäfer, MarkusGassa, AsmaeSchaiquevich, PaulaGaßler, NikolausScharovsky, Olga GracielaGaston, KevinSchattner, Mark A.Gatti, LauraScheffler, MatthiasGatto, LidiaScheie, DavidGatz, SusanneScheijen, BlancaGauchotte, GuillaumeScheipl, SusanneGaudreau, Pierre OlivierSchell, MichaelGautier, MathieuSchengrund, Cara-lynneGavard, JulieSchenke, SimoneGavazzi, FrancescaScherer, DominiqueGavert, NancyScherthan, HarryGavin, KaraScherwath, AngelaGaviraghi, MarcoScherz-Shouval, RuthGaykalova, Daria A.Schiavello, ElisabettaGazouli, MariaSchiavetti, AmaliaGe, XinSchiavina, RiccardoGe, YubinSchiavone, NicolaGeary, SeanSchiavoni, GiovannaGebauer, BernhardSchick, Joel A.Gebauer, FlorianSchierz, OliverGebbia, VittorioSchildgen, OlivierGefeller, OlafSchildgen, VerenaGeisberger, RolandSchildkraut, Joellen M.Geisler, JürgenSchilling, ClareGeismann, ClaudiaSchindlbeck, ChristianGeldmacher, DavidSchinke, Carolina D.Gelsomino, FrancescoSchinke, ThorstenGener, PetraSchiødt, Frank VinholtGenitori, LorenzoSchioppa, TizianaGennari, AlessandraSchirmer, BastianGenovesi-Ebert, FedericaSchirren, RebekkaGentilini, Oreste D.Schlack, KatrinGenuardi, ElisaSchlaepfer, IsabelGeoffrois, LionnelSchlumbrecht, MatthewGeok Hoon, LimSchmeel, Leonard ChristopherGeorgakilas, AlexandrosSchmelz, Eva M.Georgantopoulos, PeterSchmid, EviGeorges, StevenSchmid, Thomas E.Georgoulias, VassilisSchmidl, DoreenGeppert, CarolSchmidt, HenrikGérard, Jéan PierreSchmidt, ManfredGergely, LajosSchmidt, MarcusGerhard, MarkusSchmidt, Peter ThelinGerloff, DennisSchmidtke, GunterGermain, PierreSchmitt, FernandoGermanidis, GeorgiosSchmitt, NicoleGermini, DiegoSchmitz, MarcGerner, ChristopherSchmitz, UlfGerum, SabineSchmitz-Dräger, Bernd J.Geso, MoshiSchneider, GabrielaGessani, SandraSchneider-Stock, RegineGessi, StefaniaSchnütgen, FrankGessler, FlorianSchoeftner, StefanGeuna, MassimoSchoffer, OlafGevertz, JanaSchonfeld, SaraGhanim, BahilSchönthal, Axel H.Ghansah, TomarSchorb, ElisabethGhantoji, Shashank S.Schrader, JörgGhazal, HassanSchrefler, BernhardGhebeh, HazemSchrump, David S.Gherardin, NicholasSchueler, JuliaGherase, MihaiSchug, ZacharyGhiasvand, RezaSchuler, LindaGhiringhelli, FrançoisSchuler, PatrickGhodoussipour, Saum B.Schuller, Hildegard M.Ghosh, ArijitSchulte, FionaGhosh, Paramita M.Schulte, ReinhardGhosh, SouravSchulz, Wolfgang A.Ghosh, SunitaSchulze-Bergkamen, HenningGiaccone, LuisaSchuringa, Jan JacobGiacomelli, LucaSchuster, IonaGiacomelli, MichaelSchwarz, HerbertGiacomini, PatrizioSchwarzbacherová, VieraGiacopuzzi, SimoneSchwind, SebastianGiampieri, RiccardoScimeca, ManuelGianelli, UmbertoScioli, Maria GiovannaGiannella, LucaScomparin, AnnaGiannetta, ElisaSconocchia, GiuseppeGiannì, Aldo BrunoScott, AlasdairGiannouli, StavroulaScott, Jeffrey F.Giatsidis, GiorgioScott, Rodney J.Gibbons, AndreaSeano, GiorgioGibbs, BernhardSebastianelli, ArcangeloGibbs, PeterSebens, SusanneGibson, SpencerSebestyén, AnnaGiebel, SebastianSecomb, Timothy W.Giesen, NicolaSecrier, MariaGil, JustynaSedykh, SergeyGilbert, Duncan C.See, Kay ChoongGilhuijs, KennethSeger, RonyGill, HarinderSegovia, CristinaGilles, ChristineSégui, BrunoGillet, GermainSeguin, LaetitiaGillet, Jean PierreSegura, Miguel F.Gillinder, KevinSeibel, Nita L.Ginesu, GiorgioSeidl, MaximilianGiommoni, ElisaSeidlits, StephanieGiordano, AntonioSeif, Alix E.Giordano, GuidoSeith, FerdinandGiorgakis, EmmanouilSejda, AleksandraGiorgini, ElisabettaSekino, YoheiGiovannetti, ElisaSeliger, CorinnaGiovannini, GiadaSeligson, NathanGiovarelli, MirellaSelingerova, IvetaGiraldo, PilarSelivanova, Svetlana V.Girault, AlbanSelleri, CarmineGirelli, RobertoSellitto, AssuntaGiri, Anil K.Semczuk, AndrzejGirotto, StefaniaSemedo, TeresaGisselbrecht, ChristianSemenzato, GianpietroGitto, Sarah B.Sengal, AsmeromGiudice, ValentinaSengodan, SatheeshGiuffrè, MauroŠenigl, FilipGiurisato, EmanueleSenkal, CanGiustacchini, AliceSerafini, SimoneGjorgjieva, MonikaSergi, Pier NicolaGkolfakis, ParaskevasSerio, GabriellaGlantzounis, Georgios K.Sério, SusanaGlass, JonSerra, RaffaeleGlatting, GerhardSerrano-Pertierra, EstherGleeson, NoreenSerratì, SimonaGliwiński, MateuszSerša, IgorGlossop, NeilServais, StephaneGlover, AnthonySetaluri, VijayasaradhiGnanasundram, Sivakumar VadivelSethunath, VidyalakshmiGodet, YannSettembre, CarmineGodlewska, MarlenaSevenich, LisaGodlewski, JanuszSeveri, StefanoGodos, JustynaSeverin, FilippoGoel, SanjaySevigny, MaryGoemans, BiancaSeyfried, SteffenGogulamudi, Venkateswara ReddySeymour, John F.Gogvadze, VladimirSfacteria, AlessandraGokhale, PrafullaShabason, Jacob E.Gokulan, Ravindran CaspaShackelford, JuliaGoldman, RadoslavShah, Girish M.Golebiewska, AnnaShah, JainilGolias, TerezaShah, RuchiGolla, UpendarShahbaaz, MohdGöllner, StefanieShahriyari, LeiliGolubnitschaja, OlgaShaik, ShahenshaGolubovskaya, VitaShaikh, MushfiqGolumbeanu, MonicaShajahan-Haq, Ayesha N.Gomarasca, MartaShakeel, FaiyazGomes, CatarinaShamay, YosiGomes-da-Silva, Lígia C.Shammas, MasoodGómez Cerezo, Jorge FranciscoShan, YiGómez De Cedrón, MartaShankar, EswarGomez Gomez, EnriqueShankargouda, PatilGomez-Sanchez, CelsoShanmugam, VigneshGomez-Serranillos, Marta SanchezShao, JieyaGonçalves, Ana CristinaShao, LijianGonçalves, BrunoShao, YanjiaoGongora, CelineShao, Yu-Hsuan JoniGoni, FelixSharif, JafarGonnella, RobertaSharker, Shazid Md.Gonsalves, Wilson I.Sharma, AjayGonzález, ÁlvaroSharma, AnandGonzalez, AntonioSharma, Bhesh RajGonzalez, Miguel ToroSharma, GeetaGonzalez, Raul S.Sharma, NikitaGonzález, SegundoSharma, PiyushGonzález, SoniaSharma, ShwetaGonzález-Gascón Y Marín, IsabelSharonov, George VladimirovichGonzález-Sarmiento, RogelioShaw, PriyankaGonzález-Vallinas, MargaritaShehata, MohamedGoodwin, Craig M.Sheikh, NadeemGoossens, NicolasShen, Che-HungGopal, KeshavShen, GuodongGopalakrishnapillai, AnilkumarShen, ShiqianGorchakov, Andrey A.Shen, YuGorczynski, ReginaldSheng, YueGordeeva, OlgaSher, Yuh-PyngGore, Mitchell R.Sherbenou, Daniel W.Gorgulu, KivancSherif, AmirGoričar, KatjaSherwani, Mohammad AsifGorji, AliSheshadri, NamrathaGorrell, MarkShetty, MihirGorynski, KrzysztofSheu, JimGoss, Alastair N.Shi, DiGoswami, SandeepShi, Hon-YiGoto, AkiteruShi, JianGoto, TaichiroShi, KuangyuGoto, YusukeShi, LeiGotthardt, MartinShi, LewisGoulet, EricShi, MinGoussia, Anna C.Shiah, Shine-GwoGout, JohannShiba, KiyotakaGouttefangea, CecileShibata, YasushiGozzini, AntonellaShidoji, YoshihiroGrabarek, Beniamin OskarShieh, Dar-BinGrabbert, Markus TobiasShigeta, KoheiGrabowska, Anna M.Shih, Jing-WenGracia, Jose Luis PerezShiina, MarisaGraczyk-Jarzynka, AgnieszkaShimoda, MitsugiGrade, MarianShimodaira, ShigetakaGradissimo, AnaShimojo, MasahitoGraesser, FriedrichShimokawa, TsuneoGraf, Solomon A.Shimoni, AvichaiGraff, PierreShimose, ShigeoGraham, CharlotteShin, Daniel SanghoonGraham, JeffreyShin, Vivian YvonneGrana, Chiara MariaShinde, AparnaGranados-Principal, Sergio M.Shinji, KawabataGranato, MarisaShinohara, HisashiGranberg, DanShinohara, ShogoGrand, RogerShinomiya, NariyoshiGrande, FedoraShintani, YasushiGrander, ChristophShiota, SeijiGranito, AlessandroShioyama, YoshiyukiGrasa, LauraShirabe, KenGraslund, TorbjornShiradkar, RakeshGrass, G. DanielShirai, KeisukeGrassi, GabrieleShiraishi, KenshirouGrauslund, MortenShiraishi, KouyaGraves, Lee M.Shirato, HirokiGravis, GwenaelleShirole, NitinGray, Steven G.Shiue, Yow-LingGrazi, Gian LucaShivakumar, PranavGraziani, GraziaShnyder, SteveGreen, MichaelShojaei, ShahlaGregorieff, AlexShoji, SunaoGreidanus, Michiel A.Shore, NealGreither, ThomasShoshani, OferGriebenow, KaiShoumariyeh, KhalidGriffin, Robert J.Shpacovitch, VictoriaGrignani, GiovanniShramova, Elena IvanovnaGriguer, Corinne E.Shrestha, DhanGriñán-Lisón, CarmenShrestha, HridayaGrippo, Paul J.Shu, SherryGrisanti, SalvatoreShuboni-Mulligan, DorelaGrit, Jamie L.Shukla, KirtikarGrodzik, MartaShukla, SiddharthGroma, ValerijaShuo, QieGroner, BerndShurin, MichaelGronwald, JacekSibon, DavidGrösch, SabineSibson, David RossGrose, DerekSiddique, Abu BakarGross, Julia ChristinaSidhu, Stan B.Gross, Stephane R.Sieber, OliverGroßhans, JörgSiegel, PeterGrosu, Anca-LigiaSiemer, StefanGrötzinger, CarstenSiena, SalvatoreGrumati, PaoloSieni, ElisabettaGrumolato, LucaSierżęga, MarekGrüner, FlorianSifrer, RobertGrüning, Björn A.Signorelli, ChristinaGruppo, MarioSikic, DanijelGrzegrzółka, JȩdrzejSikora, EwaGrzybowska, Ewa A.Sikov, William M.Grzywa, TomaszSilic-Benussi, MicolGu, BinSillerud, Laurel O.Gu, XiaolianSilva, Ana LuísaGu, XinbinSilva, CatarinaGuadagni, FiorellaSilva-Rodríguez, JesúsGuadagni, StefanoSilver, Daniel J.Guan, FadaSilverman, Michael J.Guan, YihongSilvestri, GiovanninoGuarino, Andrea MariaSilvestri, MarcoGuarino, MariaSilvestri, ValentinaGuda, Maheedhara R.Silvestris, NicolaGudey, Shyam KumarSim, Sung HoonGueresir, ErdemSimão, André L.Gueron, GeraldineSimeone, ClaudioGuerra, CarmenSimeone, PasqualeGuerra, FloraSimionescu, NataliaGuerra, JessicaSimon, ChristianGuerra-Librero, AnaSimon, ThomasGuerriero, IlariaSimona, KranjcGuèye, PaulSimons, Brian WesleyGugasyan, RaffiSimpson, FionaGuglielmo, PriscillaSims, JenniferGugnoni, MilaSinescu, Ruxandra DianaGuh, Jih-HwaSinger, Eric A.Guha, PrasunSinger, StephanGuha, UdayanSingh, Ajay VikramGuida, MicheleSingh, Anurag K.Guidi, PatriziaSingh, ChandraGuidoboni, MassimoSingh, DeepakGuidoni, LauraSingh, NehaGuillaud, MartialSingh, S. K.Guillaume, Thierry A.Singh, Santosh KumarGuillermet Guibert, JulieSingh, SarbjitGuirouilh-Barbat, JoseeSingh, ShilpaGujar, HemantSingh, SnehaGulati, RohitSingh, Vivek KumarGuldvik, Ingrid JennySinghal, Sandeep K.Gulla, AisteSinha, Birandra K.Gulve, NitishSinha, DebottamGulyaeva, Lyudmila F.Sinha, DivyaGunaje, JayaramaSinha, RaghuGundlach, Jan-PaulSini, Maria CristinaGuo, LilongSinnberg, Tobias W.Guo, SiqiSioud, MouldyGuo, ZengliSipos, FerencGuo, Zong ShengSippl, WolfgangGuolo, FabioSiraj, AfjalusGupta, AjaySiravegna, GiuliaGupta, MohitSirinian, ChaidoGupta, Ramesh C.Sirvent, AudreyGupta, RisheinSitar, Daniel S.Gupta, RomiSitbon, MarcGupta, SanjaySitia, LeopoldoGupta, SeemaSiu, Michelle K. Y.Gupta, Vijayalaxmi G.Sivakumar, SushamaGupta, Vineet K.Siwko, StefanGupta, YashSjöberg, Rickard L.Gurnari, CarmeloSkardelly, MarcoGurney, HowardSkiecevičienė, JurgitaGursky, JanSkoda, JanGust, Kilian MartinSkonieczna, MagdalenaGuth, AmandaSkov, VibeGutierrez, Linda S.Skrap, MiranGutiérrez, María LauraSkręt, AndrzejGutierrez-Aguilar, ManuelSkrzypczak, MaciejGuttery, David S.Skrzypek, KlaudiaGutzmer, RalfSkvortsova, Ira-IdaGuyon, LaurentSlade, NedaGuyot, BorisSlobodin, BorisGuyot, ErwanSlominski, AndrzejGyllensten, UlfSlomovitz, Brian M.Gyulai, RollandSłowińska-Klencka, DorotaHa, JeonghoonSmaglo, Brandon G.Ha, Min-wooSmahel, MichalHa, Yun-SokSmetana, KarelHaag, FriedrichŚmieszek, AgnieszkaHaag, MathiasSminia, PeterHabibollahi, PeimanSmith, Deanna S.Hacker, NevilleSmith, EricHacker, UlrichSmolarczyk, RyszardHackl, ChristinaSmolle, JosefHackl, HubertSmrž, DanielHaderlein, MarlenSnijders, Tom J.Hadidi, Samer AlSnow, StephanieHadler-Olsen, Elin SynnøveSnyder, ChristopherHaffner, MichaelSo, JonathanHäfner, NormanSoares, PaulaHagemann, Anja I. H.Soave, ArminHägerling, RenéSobas, MartaHaglund, FelixSobhani, NavidHahn, OliverSobiak, JoannaHahne, JensSobolewski, CyrilHahne, MichaelSobrinho-Simões, ManuelHaier, JörgSocea, BogdanHájek, RomanSocha, MałgorzataHaj-Hosseini, NedaSocorro, SilviaHalaban, RuthSoddu, SilviaHałas-Wiśniewska, MartaSodergren, Samantha C.Haldorsen, Ingfrid S.Soejima, ToshinoriHalicek, Martin T.Soffietti, RiccardoHalkett, GeorgiaSogawa, RintaroHall, Per F. L.Sohrabi, AmirHallaert, GiorgioSoibam, BenjaminHallden, GunnelSolana, RafaelHam, Won-SikSolanki, SumeetHamada, ShinSolbiati, LuigiHamamoto, RyujiSolé Cañadas, CarlaHamanishi, JunzoSolimando, Antonio G.Hamburger, Anne W.Solinas, GiulianaHammad, Mohamed AdelSolip, ParkHammer, ElkeSolli, PiergiorgioHammill, JaredSoltész, BeátaHammoud, Ghassan M.Somasundaram, AshwinHamzeh-Cognasse, HindSon, JaekyoungHan, ChialiSondak, VernonHan, DongSondarva, GautamHan, HongweiSone, HidekoHan, IlkyuSong, Chang W.Han, Ki-HoSong, ChanghoonHan, YanhuiSong, GuishengHan, YoungminSong, JaewhanHan, YuyanSong, MinjungHanafy, Nemany Abdelhamid NemanySong, XinniHancock, SarahSoni, AashishHanda, HiroshiSonis, StephenHanin, LeonidSonowal, HimangshuHanke, TomasSoon, HongHanker, AriellaSoond, Surinder M.Hanks, Brent A.Soons, ZitaHanley, ChirstopherSørensen, Anne VestHannoush, Zeina CarolinaSorensen, Poul H.Hanoun, MaherSorigué, MarcHansen, Carsten GramSorolla, Maria AlbaHansen, Charlotte ToftmannSoror, Tamer Y.Hansen, Hinrich P.Sorrenti, SalvatoreHansen, Jakob WernerSorrentino, RosalindaHansen, Torben FrøstrupSosnowska-Pasiarska, BarbaraHappel, ChristianSossey-Alaoui, KhalidHapuarachchige, SudathSotirchos, VlasiosHaque, Muhammad R.Sotomayor, Camilo G.Haque, ReinaSouers, AndrewHarada, MamoruSouglakos, JohnHarada, TakeshiSoulen, Michael C.Harada, ToshiyukiSounni, Nor EddineHarashima, NanaeSousa, AngelaHarbison, R. AlexSouthall, NoelHarbottle, RichardSouwer, Esteban T. D.Harder, AnjaSp, NipinHardt, JuliaSpaargaren, MarcelHardt, OlafSpadarella, GaiaHardtke-Wolenski, MatthiasSpagnolo, FrancescoHareendran, SangeethaSpałek, MateuszHarisinghani, MukeshSpałek, Mateusz JacekHarling, LeanneSpan, PaulHarrell, J. ChuckSpanakis, MariosHarrer, DennisSpanheimer, PhilipHarrington, BrittneySpathis, ArisHarris, Brent T.Speeckaert, MarijnHarrison, David J.Sperling, AdamHärteis, SilkeSperti, CosimoHartl, Dana M.Spiazzi, LuigiHartley, AndrewSpindler, Karen Lise GarmHartman, JohanSpinelli, LauraHartman, MariuszSpinnato, PaoloHartmann, DanielaSpirina, Liudmila V.Hartmann-Johnsen, Olaf JohanSpoerri, LoredanaHartsough, EdwardSpoletini, GabrieleHarvey, James R.Sprave, TanjaHasanzadeh Kafshgari, MortezaSprengers, DaveHasegawa, ShinyaSpring, KevinHasegawa, SumitakaSreedharan, SreejeshHashida, NoriyasuSrikanthan, AmirrthaHashimoto, DaisukeSrivastava, AnkitHashimoto, KazuhikoSrivastava, AnujHasipek, MetisSrivastava, Swayam PrakashHasoňová, LucieSroka, RonaldHassan, MohamedStaber, Philipp BernhardHassan, Sherif T. S.Stack, SharonHasse, SybilleStadlbauer, AndreasHasselgren, Per OlofStafford, Francis WilliamHata, MasaharuStål, PerHatakeyama, HirotoStålberg, PeterHatano, KojiStalpers, LukasHatano, YuichiroStanganelli, IgnazioHato, TaiStanislawska-Sachadyn, AnnaHattori, NaokoStannula, MartinHau, PeterStanzione, ArnaldoHaug, AlexanderStarzyńska, AnnaHaugk, BeateStathopoulos, ConstantinosHaugnes, Hege SagstuenStati, ValeriaHaumaitre, CécileStauber, RolandHauptmann, SteffenStaudenherz, AntonHauser, HansjoergStawski, RobertHauser, PeterStecca, TommasoHaveman, Lianne M.Steckiewicz, KarolHawkridge, Adam M.Steeghs, NeeltjeHay, JenniferSteenblock, CharlotteHayasaka, HarukoSteenbrugge, JonasHayashi, KatsuhiroStefano, AlessandroHayashida, KenjiStefanowicz, JoannaHaybaeck, JohannesStefanska, AnnaHaynes, JenniferSteinbach, JoachimHayward, Mary-KateSteinbrunn, TorstenHazama, ShyouichiSteinemann, DorisHazawa, MasaharuSteinkamp, Mara P.Hazlehurst, Lori A.Stella, AlessandroHe, JinStella, Giulia M.He, YazhouStella, StefaniaHe, YukaiStelmes, Jean-JacquesHeakal, YasserStemmler, MarcHealey, GarethStenman, AdamHebeda, Konnie M.Stenzl, ArnulfHeckl, DirkStepanenko, Aleksei A.Hee-chul, ParkStepanov, Alexey V.Heeke, SimonStephen, Andrew G.Heer, RakeshStepp, HerbertHegde, BinduStergios, KonstantinosHeger, UlrikeSteri, VeronicaHeiblig, MaelSterpetti, Antonio V.Heigener, David F.Stewart-Ornstein, JacobHeinzmann, DavidStirke, ArunasHeitmann, Jonas S.Stobiecka, MagdalenaHejnar, JiriStock, ChristianHelen Jane, BoyleStockmann, ChristianHeller, GerwinStøer, Nathalie C.Heller, RichardStoian, DanaHelms, VolkhardStoklosa, TomaszHenderson-Jackson, EvitaStoppelli, Maria PatriziaHendriks, ManyaStorey, KathleenHeng, Henry H.Störmann, SylvèreHéninger, ErikaStovell, MatthewHenkenberens, ChristophStrand, ElinHennig, EwaStrano, SabrinaHennkens, Heather M.Strati, AretiHenry, ClaireStratikos, EfstratiosHenry, Ryan A.Straus, David J.Henry, YvesStravopodis, Dimitrios J.Henry-Amar, Michel C.Streba, Costin TeodorHenson, JeremyStrigari, LidiaHeras, Sara M.Strippoli, RaffaeleHerbig, MaikStröbel, PhilippHerbreteau, GuillaumeStrocchio, LuisaHerbst, AllenStrowitzki, MoritzHerbst, AndreasStrub, ThomasHercbergs, AleckŠtrumfa, IlzeHerfarth, Klaus K.Strus, MagdalenaHermann, R. M.Strzelczyk, JoannaHermouet, SylvieStuart, HeatherHernandez-Alcoceba, RubenStuppia, LiborioHernãndez-Bronchud, Miguel H.Sturtzel, CaterinaHernández-Rivas, José-ÁngelStüssi, GeorgHernández-Sánchez, MaríaSu, Lydia Min-YingHerndler-Brandstetter, DietmarSu, RuiHernes, Eivor H.Su, Shih-binHerr, IngridSu, Wen-PinHerraez, ElisaSu, XinmingHerrera, BlancaSu, Ying-HsiuHerreros-Pomares, AlejandroSuarez-Cunqueiro, María MercedesHersey, PeterSubirán, NereaHerskind, CarstenSuckow, MarkHervé, TécherSuda, KenichiHervouet, EricSudbery, IanHerwig-Carl, Martina C.Suehara, YoshiyukiHester, JoannaSuematsu, MakotoHettie, KennethSugaya, MakotoHeumann, RolfSugie, TomoharuHeuskin, Anne-CatherineSugimoto, MitsushigeHicks, RodneySugimura, Rio RyohichiHicks, Wesley L.Suh, Chong HyunHickson, IanSuh, YunsuhkHier, MichaelSuhail, YasirHigano, Celestia S.Suijkerbuijk, KarijnHigurashi, TakumaSukhanova, Mariya V.Hijiya, NobukoSukhorukov, VladimirHilakivi-Clarke, LeenaSukowati, Caecilia H. C.Hill, MarkSulová, ZdenkaHill, MichelleSummers, Yvonne JaneHill, RebeccaSun, DuanpingHillmer, Axel M.Sun, JianminHiltbrunner, StefanieSun, JingHimoto, TakashiSun, RamonHindié, ElifSun, YilunHingorani, Dina V.Sun, YinHinkley, LeightonSun, ZhonghuaHirano, HidekazuSunami, YoshiakiHiraoka, NobuyoshiSundararajan, KousikHirata, HiroakiSundararajan, VigneshHirata, KenjiSundín, Anders E.Hirohashi, YoshihikoSundvall, MariaHironaka, ShuichiSung, Kyung HyunHirose, YoshinobuSung, Wen-WeiHitt, RicardoSupernat, AnnaHiwasa, TakakiSurdutovich, EugeneHjerpe, AndersSureda, AnnaHo, Cheng-MawȘurlin, Valeriu MarinHo, ChengyingSurov, AlexeyHo, Ivy A. W.Suryawanshi, AmolHo, James Chung-manSuso, Else Marit InderbergHo, Sheng-YowSutherland, KateHobbs, Gabriela SorianoSuwan, KeittisakHochwald, Steven N.Suzuki, HajimeHodny, ZdenekSuzuki, KazuhitoHodson, DanielSuzuki, ShinichiHoefler, GeraldSvahn, Tony MartinHoffmann, CarolineSvendsen, Lars BoHoffmann, FranziskaSverdlov, AaronHoffmann, MarkusSverrisdóttir, EvaHofmann, PeterSwain, Sandra M.Hogan, Andrew E.Swami, UmangHohaus, StefanŚwierczewska, MonikaHoheisel, Jörg D.Swijnenburg, R. J.Hohenberger, PeterSykiotis, Gerasimos P.Hohenegger, MartinSzabo, IldikoHohmann, TimSzanto, IldikoHöhne, JuliusSzarvas, TiborHojan, KatarzynaSzaryńska, MagdalenaHojjat-Farsangi, MohammadSzatmári, DávidHokamp, Nils GroBeSzeliga, MonikaHolmager, PernilleSzijan, IreneHolmberg, Carina I.Szilágyi, MelindaHölscher, TobiasSzmajda-Krygier, DagmaraHöltke, CarstenSzolnoky, GyőzőHolubekova, VeronikaSzoor, ArpadHolubova, MonikaSzturz, PetrHolzmann, KlausSzuhai, KarolyHombach, Andreas A.Szumera-Cieckiewicz, AnnaHombach-Klonisch, SabineSzyc, ŁukaszHonda, TomoyukiSzymanski, WiktorHonegger, JuergenSzymczak-Pajor, IzabelaHoneywell, Richard J.Tabarkiewicz, JacekHong, Andrew L.Tabatabai, GhazalehHong, ChangwanTabish, Tanveer AhmadHong, Hyun SookTaboni, StefanoHong, Min HeeTacconelli, StefaniaHonoré, BentTada, ToshifumiHooda, JagmohanTafuto, SalvatoreHoogwater, FrederikTaghizadeh, HosseinHooper, Douglas C.Tagliabue, EldaHooper, John D.Tagliabue, MartaHöpfner, MichaelTagliati, CorradoHöppner, JensTaieb, JulienHorak, PeterTakabatake, KiyofumiHoreweg, NandaTakada, YasutsuguHorger, MariusTakagi, KiyoshiHorinouchi, HidehitoTakahama, TakayukiHorn, Lucas A.Takahashi, ChiakiHornak, Joseph P.Takahashi, HiroyukiHornsveld, MartenTakahashi, MamoruHorsky, JiřiTakahashi, ShyunjiHorstmann, MarcusTakai, AtsushiHorvath, AneliaTakam Kamga, PaulHorvath, RobertTakamaru, HiroyukiHoshino, DaisukeTakaori-Kondo, AkifumiHoskins, ClareTakasawa, KenHossain, Kaniz Fatima BinteTakashi, SekiyaHosui, AtsushiTakayama, KenichiHottinger, Andreas F.Takeda, HiroyukiHou, Hsin-AnTakeda, MasayukiHou, JueTakeda, ToshikazuHouben, RolandTakei, HiroyukiHoudek, MatthewTakenaka, MamoruHouse, CarrieTalar-Wojnarowska, RenataHouse, ImranTalesa, Vincenzo NicolaHoushmand, MohammadTallet, Agnès V.Houtman, JonTalos, FlaminiaHouvenaeghel, G.Talukdar, Fazlur RahmanHoving, EelcoTalukdar, SarmisthaHoy, AndrewTam, Shing YauHrstka, RomanTam, Shing Yau MarcoHrvat, AntonioTamagno, IlariaHsiao, Hui-HuaTamaian, RaduHsiao, Kuei-YangTambuwala, MurtazaHsiao, Sheng-MouTampe, BjörnHsiao, YungchinTamura, HidetoHsieh, Chia-ChienTamura, MasaakiHsieh, Chien-MingTamura, RyotaHsieh, Ming-ChingTamura, YasuakiHsieh, Ming-LiTamvakopoulos, ConstantinHsieh, Pei-LingTan, KarenHsieh, Tsung-HanTan, LiHsu, FeitingTan, Nguan SoonHsu, Fei-TingTan, Tuan ZeaHsu, Kai-WenTan, YutingHsu, Keng-FuTan, ZhengguoHsu, Ping-ChihTanabe, HirokiHsu, Shih-HsienTanabe, ShihoriHsu, Shu-ChingTanaka, NobuyukiHsu, SigmundTanaka, YukaHsu, Tsung-ITanda, Maria LauraHsu, Wen-MingTang, Kwan HoHsu, Ya-LingTang, Patrick Ming-KuenHsu, Yi-ChiungTang, YutingHu, BaoliTangherloni, AndreaHu, JackTani, KenzaburoHu, JenniferTanic, NikolaHu, TaoboTanino, HirokazuHu, XiaowenTanioka, MakiHuang, HaiqiuTaniwaki, MasafumiHuang, HaoTaniyama, DaikiHuang, Huang ChiaoTantos, ÁgnesHuang, Huey-ChunTaraboletti, GiuliaHuang, HuocongTarallo, RobertaHuang, JunfengTarantino, GiovanniHuang, Kai WenTarn, Woan-YuhHuang, Paul Li-HaoTarnowski, MaciejHuang, Shiang-FuTartaglia, FrancescoHuang, Shin-MingTartarone, AlfredoHuang, Shu-PinTartey, SarangHuang, Szu-WeiTartour, EricHuang, Wen-ChinTaschner-Mandl, SabineHuang, XiaohuTashima, ToshihikoHuang, XudongTashireva, Liubov A.Huang, Yi-HsiangTashiro, HarukoHuang, YingTatić, SvetislavHuangFu, Wei-ChunTátrai, PéterHuber, MagdalenaTaube, JosephHuber, PeterTaubenberger, AnnaHübner, RalphTaulier, NicolasHuda, NazmulTavernier, JanHudler, PetraTaylor, Justin W.Huebbers, Christian U.Taylor, MatthewHuebner, HannaTaylor, Renea A.Huen, Michael Shing YanTaylor, VerdonHughes, KevinTazzari, MarcellaHughes, MichaelTazzioli, GiovanniHugo, WillyTchoghandjian-Auphan, AurélieHuidobro, CovadongaTebbi, Cameron K.Hulin, AnneTeicher, Beverly A.Hulleman, EstherTeijeira, ÁlvaroHulshof, Maarten C. C. M.Teisseyre, AndrzejHumphries, Jonathan D.Teixidó, CristinaHüneburg, RobertTejero, JesúsHung, Chin-ChuanTellez Gabriel, MartaHung, Ming-SzuTellier, MichaelHung, Shih-KaiTelukutla, Srinivasa ReddyHung, Yao-ChingTemel, SehimeHung, Yin PunTemraz, SallyHunter, Anthony MichaelTen Hacken, ElisaHunter, BethTenenbaum, ScottHunter, ZacharyTeng, Chieh-LinHuntington, Nicholas DavidTeng, JianHuntosova, VeronikaTeng, WeiHupe, Marie C.Teoh-Fitzgerald, MelissaHur, HoonTerai, MizueHurbin, AmandineTerao, MinekoHusnjak, KoraljkaTeras, Lauren R.Huss, RalfTerazono, HideyukiHussaini, HaizalTeresa, CalimeriHutchinson, John N.Terp, Mikkel GreenHuvenne, WouterTerry, Paul D.Hwang, Sung IlTerry, SamanthaHwangbo, CheolTerry, StéphaneHwu, Yueh-JuenTerry, Stephen JHyun, Sang-HwanTerstappen, LeonIaccino, EnricoTerzuoli, LuciaIachina, Maria K.Tesson, MathiasIacobucci, IlariaTessoulin, BenoitIacovelli, RobertoTestori, Alessandro A. E.Iannitto, EmilioTeterycz, PawełIatí, GiuseppeTeusch, NicoleIavarone, FedericaThakur, AbhimanyuIbáñez-Costa, AlejandroThakur, ShilpaIbbs, MatthewThalhammer, TheresiaIbrahim, AbdallaThangavel, ChellappagounderIbrahim, Sherif AbdelazizTharehalli, UmeshIdoate, MiguelTheil, GeritIerardi, EnzoTheillet, CharlesIgaz, PeterThellung, StefanoIglesias, EmiliaThemistocleous, MariosIglesias-Ara, AinhoaThevenod, FrankIgnatius, MyronThevenot, Paul T.Ignee, AndreThijssen, RachelIhle, AndreasThomas, GijuIkematsu, HiroakiThomassen, MadsIkesue, HiroakiThomsen, Caroline Emilie BrennerIkonomopoulou, MariaThomsen, Martin K.Illei, Peter B.Thomsen, ThomasIllert, LenaThong, Melissa Suk YinIlling, Patricia T.Thorenoor, NithyanandaIlonen, Ilkka K.Thurber, Greg M.Imai, ChihayaThurston, David E.Imai, YasuoThuru, XavierImamura, MasahiroThyagarajan, BharatImanirad, ImanTiberio, Guido Alberto MassimoImig, JochenTicconi, CarloIn ′t Veld, Sjors G. J. G.Tiller, JaneIn ′t Zandt, ReneTilvawala, RonakInama, MarcoTimchenko, NikolaiInamdar, SumantTimo, CarpénInderberg, Else MaritTimofeev, OlegIndini, AliceTimoshenko, ViktorIndraccolo, StefanoTinari, NicolaInfante, MarTinelli, AndreaInga, AlbertoTing, Wen-ChienIngram, NicolaTinguely, MarianneIngrosso, GianlucaTiribelli, ClaudioInno, AlessandroTiribelli, MarioInnominato, PasqualeTiriveedhi, VenkataswarupInoue, TakahiroTischler, ArthurInyushin, Mikhail Y.Tittarelli, AndrésIoffe, YevgeniyaTixeira, RochelleIommelli, FrancescaTixier, FlorentIrace, CarloTiziani, StefanoIrini, EvnouchidouTjahyono, Daryono HadiIriuchishima, HironoTobiume, KeiIrjala, HeikkiToda, TakashiIsayama, HiroyukiTodaro, MatildeIsemura, MamoruToden, ShusukeIshiai, MasamichiTodoerti, KatiaIshibashi, ToyotakaTofanelli, MargheritaIshida, FumihiroTognolatti, PieroIshida, TadaoToh, Tan BoonIshido, KeinosukeTohru, MorisadaIshihara, KatsuhikoToillon, Robert-AlainIshihara, SeiichiroToivanen, RoxanneIshikawa, EiichiTokino, TakashiIshikawa, EriTokumaru, SunaoIshikawa, HitoshiTokumaru, YoshihisaIshikawa, MitsuyaTokunaga, HidekiIshikawa, ToruTokushige, KatsutoshiIshitsuka, YousukeToledano, Michel B.Ishiwata-Endo, HirokoToledo, FranckIshiyama, HiromichiTolosano, EmanuelaIshizawa, TakeakiTomaiuolo, RossellaIshizuka, MitsuruTomasetto, CatherineIslam, SharifulTomasetto, Catherine-LaureIsohashi, FumiakiTomasi, Maria LaudaIsola, MiriamTomašič, TihomirIssels, Rolf DieterTomasini, RichardItabaiana, IvaldoTomasson, MichaelItabashi, MichioTomaszkiewicz, MartaItälä-Remes, MaijaTometich, DanielleItaliano, AntoineTomić, JosipItami, JunTominaga, NaoomiItamochi, HiroakiTomoaki, OkimotoItkonen, HarriTomuleasa, CiprianIto, HiromichiTonacci, AlessandroIto, TakamichiTonelli, FrancescoItuarte, PhilipTong, JessicaIwamoto, HidekiTong, ManIwamoto, ShotaroTordai, AttilaIwanicki, MarcinTordjman, Karen M.Iwaszkiewicz-Grzes, DorotaTorigoe, ToshihiroIwata, TakehiroTorimura, TakujiIzdebska, MagdalenaTornesello, Anna LuciaIzzotti, AlbertoTornillo, GiusyJabłońska, BeataTornin, JuanJackson, JamesTorrisani, JérômeJackson, Michael A.Torrisi, RosalbaJacob, SheebaTorstein, MelingJacobsen, AndersToruner, Gokce AltayJacqmin, HuguesToschi, ElenaJacquelot, NicolasToshito, ToshiyukiJafari, Nancy NajmehTosti, GiulioJäger, RolandToth, RekaJaggi, MeenaToti, PaoloJagiełło-Gruszfeld, AgnieszkaTotonchy, JenniferJahangiri, LeilaTotta, PierangelaJahn, ArneTournigand, ChristopheJain, SuneilToutain, JérômeJaiswal, Ashvin R.Touw, Ivo P.Jaiswal, DeepikaTovoli, FrancescoJakopovic, MarkoTownsend, JulieJakšić, DanielaToyama-Sorimachi, NorikoJakubek, MilanTrader, DarciJakubowska, Monika A.Trainer, Alison H.Jalkanen, SirpaTrama, AnnalisaJamar, FrançoisTrapani, DarioJames, Claire D.Traub, FrankJamitzky, SilkeTraverso, AlbertoJan, Ming-ShiouTreacy, OliverJanczar, SzymonTreglia, GiorgioJanda, ElzbietaTrembley, JaneenJandrot-Perrus, MartineTretter, LaszloJanecki, MarcinTrevisani, FrancoJané-Salas, EnricTrevisi, GianlucaJang, Hyun-jongTriche, TimothyJang, Sung-WukTrikalinos, NikolaosJaníková, AndreaTrimboli, PierpaoloJanikowska, GrażynaTriner, DanielJankovic, MomciloTripathi, MadhulikaJanmaat, Vincent T.Tripathi, RakshamaniJänne, Olli A.Tripon, FlorinJanody, FlorenceTrivedi, RachanaJanowski, MiroslawTroadec, Marie-BérengèreJansen, C. A. (Christine)Troconiz, Iñaki F.Jansen, GerritTrojani, AlessandraJansen, Yanina Jeanne Leona.Troschel, FabianJanssen, EmielTroschel, Fabian MartinJanssen, NicoleTrotta, RossanaJansson, Patric J.Trovato, MariaJanuszkiewicz-Lewandowska, DanutaTrufero, Javier MartínezJarząb, BarbaraTruillet, CharlesJassim, Sarmad H.Truksa, JaroslavJaukovic, AleksandraTsagozis, PanagiotisJavadian, PouyaTsai, Chi-NeuJavaheri, TaherehTsai, Hung-WenJaworek- Korjakowska, JoannaTsai, I-LinJaworek, JolantaTsai, Jo-TingJayaprakash, PriyamvadaTsai, Ping-HsingJaye, David L.Tsai, Shih-changJazayeri, Seyed BehzadTsai, Wan-ChiJazirehi, Ali R.Tsai, Wei-LunJeffers, Kate D.Tsai, Ya HuiJelinek, TomasTsai, Yuan-ChenJenkins, Russell W.Tsakalof, AndreasJennifer, SunTsatsakis, AristidisJeon, Min JiTsaur, IgorJeong, Kyoung-sookTschernig, ThomasJeong, SeriTseng, Chun-ChihJeong, Seung-YongTseng, JenchihJeschke, UdoTseng, Jen-ChihJesenko, TanjaTseng-Rogenski, Stephanie S.Jesse, Christopher M.Tsingotjidou, AnastasiaJeucken, AikeTsirigotis, PanagiotisJi, PengTsoi, HoJi, ZhichengTsoli, MariaJian, XingTsougos, IoannisJiang, DaweiTsuboi, MayoJiang, LiTsubota, AkihitoJiang, Lin-HuaTsuchiya, HiroyukiJiang, Shih ShengTsuchiya, TomoshiJiang, XiaodongTsuchiya, YuichiJiang, YongTsuji, Atsushi B.Jiménez-González, GemaTsuji, TakahiroJiménez-Luna, CristinaTsujikawa, TakahiroJimi, EijiroTsukada, HideoJin, Dong-HoonTsukahara, ToshifumiJin, JiefuTsuneoka, MakotoJin, WeilinTsurusaki, MasakatsuJin, ZhaohuiTsuzuki, ToyonoriJochmanová, IvanaTucci, MarcoJochum, ChristophTucci, PaolaJoh, Richard InhoTucker, MatthewJohanns, Tanner M.Tuech, Jean-JacquesJohansson, UlrikaTulchinsky, EugeneJohnen, GeorgTummala, PavanJohnson, Christopher M.Turashvili, GulisaJohnson, James E.Turco, Luigi CarloJohnson, JeffTurcotte, SimonJohnson, Jennifer M.Turdo, AliceJohnson, Nathalie A.Turesson, Ingela E.Jöhrens, KorinnaTuretta, MatteoJolly, Mohit KumarTurhan, Ali G.Jomaa, Mona K.Turpin, AnthonyJonassen, ChristineTuynman, JurriaanJones, BleddynTvrdik, PetrJones, KennethTyagi, AshishJönsson, Jenny-MariaTyan, Yu-ChangJordan, Bénédicte F.Tylkowski, BartoszJørgensen, HeatherTysarowski, AndrzejJoseph, ChitraTzanakakis, GeorgeJoshi, GauravTzang, Bor-ShowJoshi, Molishree UmeshTzankov, AlexandarJoshi, SonaliTzelepi, VasilikiJosse, ClaireTzeng, I-ShiangJost, EdgarTziatzios, GeorgiosJou, Yuh-shanUchiumi, FumiakiJoura, Elmar A.Udager, Aaron M.Jourdain, AlexisUddin, ShahabJozwik, AgnieszkaUehara, KeiichiroJozwik, MarcinUetake, HiroyukiJu, XiangqunUgel, StefanoJud, Sebastian M.Ugga, LorenzoJuette, HendrikUgolini, ClaraJuhasz, KataUhl, EberhardJuhlin, ChristoferUkidve, AnvayJulián, EstherUm, Hong-DuckJulià-Sapé, MargaridaUmansky, Victor Y.Jung, Chan KwonUmansky, ViktorJung, Ji HoonUmemori, JuzohJung, JooheeUmemura, AkiraJung, KeehoonUmemura, TsukuruJung, KlausUng, Choong YongJung, Yuh-SeogUngefroren, HendrikJunker, KerstinUngureanu, Bogdan SilviuJuozaitytė, ElonaUpadhyaya, JasbirJurj, Ancuţa MariaUprety, DipeshJurkovicova, DanaUrbanek-Trzeciak, Martyna O.Kabe, YasuakiUrbanelli, LorenaKabir, Mohammad FaujulUrbaniak, AlicjaKabziński, JacekUrbanska, Edyta MariaKacew, Alec J.Urbanucci, AlfonsoKaczmarek, RadoslawUrbschat, Steffi M.Kae, NakamuraUreshino, HiroshiKaesmann, LukasUrsic-Bedoya, JoséKaewkhaw, RossukonUrsino, StefanoKagamu, HiroshiUrso, EmanueleKagey, JacobUshmorov, AlexeyKahlert, UlfUsui, TatsuyaKaigorodova, EvgeniyaUygun, Basak E.Kaira, KyoichiUyl-de Groot, Carin A.Kairemo, KaleviUysal-Onganer, PinarKajihara, MikioUzunoglu, Faik G.Kajimoto, ShinjiVacca, AngeloKajtár, BélaVaccari, SamueleKakehashi, AnnaVachtenheim, JiriKakizawa, NaoVaclavikova, RadkaKakkassery, VinodhVaddi, PrasannaKalainayakan, Sarada PreetaVaickus, Louis J.Kalamarides, MichelValachis, AntonisKalasauskas, DariusValasoulis, GeorgeKalathil, SureshValencia, KarmeleKalathiya, UmeshValente, GuidoKalayda, GannaValentijn, L. J.Kalb, ReinhardValentiner, UrsulaKalinova, MarketaValentini, ValentinoKalirai, HelenValentini, VirginiaKaliszewski, KrzysztofValeryia, Valeryia MikalayevaKalita-de Croft, PriyakshiValiante, SalvatoreKaljunen, HeidiValiveti, ChaitanyaKállay, EniköVallacchi, VivianaKallendrusch, SonjaValle, Marcos EduardoKaller, MarkusVallera, DanielKallergi, GalateaVallet, SoniaKallioniemi, ElisaValletti, AlessioKalthoff, HolgerValteau-Couanet, DominiqueKaltsas, GregoryValtorta, SilviaKaltschmidt, BarbaraValverde, ClaudiaKałuża, AnnaVamvakaris, IoannisKamada, YoshihiroVan Bokhoven, AdrieKamensek, UrskaVan Dam, Peter A.Kameyama, KaoriVan Dam, R. MichaelKamijo, TakehikoVan De Sande, Michiel A. J.Kamimura, HiroteruVan De Ven, RienekeKamimura, KenyaVan De Wiele, ChristopheKaminska, BozenaVan Den Bergh, HubertKammala, Ananth KumarVan Den Brekel, Michiel Wilhelmus MariaKampen, KimVan Den Heuvel, MichelKamphues, CarstenVan Den Tol, Monique PetrousjkaKamran, MohammadVan Der Hage, Jos A.Kanagasabai, ThanigaivelanVan Der Meel, RoyKanapin, AlexanderVan Der Post, Rachel S.Kanaujiya, JitendraVan Der Velden, JacobusKanayama, KojiVan Der Wekken, AnthonieKanda, TatsuoVan Deurzen, Carolien H. M.Kandasamy, PalanivelVan Dyk, MadeleKandpal, ManojVan Gompel, Jamie J.Kaneda, AtsushiVan Gool, Stefaan W.Kaneda, MasahiroVan Griethuysen, JoostKanegasaki, ShiroVan Heetvelde, MattiasKaneko, SyuzoVan Hemelrijck, MiekeKanellakis, Nikolaos I.Van Houtte, PaulKanemura, NobuhiroVan Kempen, LeonKanemura, YonehiroVan Leeuwen, Frank N.Kang, Chang MooVan Leeuwen, Ton G.Kang, ChangSunVan Lith, SanneKang, DongchulVan Moorselaar, Reindert Jeroen A.Kang, Dong-WooVan Noorden, Cornelis Johannes ForrindinisKang, EunjuVan Praet, CharlesKang, Jae SeungVan Prooijen, MoniqueKang, Koo JeongVan Schil, PaulKang, KyuhoVan Spriel, AnnemiekKang, Min KyuVan Tine, BrianKang, Tae HyunVan Tine, Brian AndrewKang, YuVan Veldhuisen, EranKanginakudru, SriramanaVan Veldhuizen, PeterKanis, Margaux JennaVan Wely, KarelKant, ShivaVan Werkhoven, ErikKao, Chien-ChangVan Wilpe, SandraKao, Shao-HsuanVan Zandwijk, NicoKaochar, SalmaVandamme, TimonKapischke, MatthiasVandenBussche, Christopher J.Kaplan, David E.Van-Dijk, JulietteKaplan, Michael J.Vangipurapu, RajanikanthKappler, MatthiasVanni, GianlucaKapral, MałgorzataVannier, MichaelKaracosta, Loukia G.Vannini, EleonoraKarakaidos, PanagiotisVaquero, JavierKarakas, CansuVaradaraj, ArchanaKarakatsanis, AndreasVaraljai, RenataKaramouzis, MichalisVaramini, PegahKarantanos, TheodorosVarga, GeorgKaravitakis, MarkosVargas-Parra, GardeniaKarin, NathanVargova, KarinaKarnati, SrikanthVarkaris, AndreasKarousou, EugeniaVarlotto, John MichaelKarow, AxelVasaikar, Suhas V.Karpathiou, GeorgiaVasan, Nilesh R.Karpel-Massler, GeorgVasavada, BhavinKarpiński, Tomasz M.Vasko, Vasyl V.Karpov, DmitryVásquez-Gómez, JaimeKarras, PanagiotisVassos, NikolaosKarreman, Matthia A.Vasuri, FrancescoKarsak, MelihaVaubel, RachaelKarthikeyan, MythreyeVaupel, PeterKartsonaki, ChristianaVazana, UdiKasahara, NoriyukiVecchio, Giada MariaKashima, ShinVeenland, JifkeKashino, GenrouVega, Francisco M.Kashiwagi, ShinichiroVega-Benedetti, Ana FlorenciaKashofer, KarlVegran, FrederiqueKasiotis, KostasVekariya, RakeshKasman, LauraVelegzhaninov, Ilya O.Kasperkiewicz, PaulinaVelez-Cruz, RenierKasprzak, AldonaVelikova, TsvetelinaKasymjanova, GoulnarVelliou, EiriniKasza, AnetaVelotti, NunzioKasztelan-Szczerbińska, BeataVenanzi, MarianoKataoka, NaoyukiVenditti, AdrianoKataoka, TatsukiVenesio, TizianaKatapodi, Maria C.Venkatadri, RajkumarKatarzyna, MalarzVenner, Christopher PaulKatayama, Maria Lucia HirataVenneti, SriramKatira, ParagVens, ConchitaKato, KatsuhikoVenturelli, SaschaKato, KenVenugopalan, AbhilashKato, Takamitsu A.Verderio, ClaudiaKato, TomoyasuVerdon, Daniel JohnKatsanis, EmmanuelVered, MarilenaKatsila, TheodoraVergara, DanieleKatsuta, ErikoVergez, FrançoisKatzenellenbogen, RachelVerheij, JoanneKauppila, Joonas H.Verheij, MarcelKaur, KawaljitVerheijen, ReneKaur, SukhbirVerhoeyen, ElsKaur, TanpreetVerma, Navin KumarKaushik, GarimaVerma, ShivKaushik, Nagendra KumarVerma, Shiv PrakashKawabata, HideakiVermassen, TijlKawabata-Iwakawa, ReikaVermeij, Wilbert P.Kawagishi, NaokiVermeulen, AnKawaguchi, NanakoVermijlen, DavidKawai, ManabuVermorken, Jan B.Kawai, TatsuyaVernieri, ClaudioKawaida, MihoVernuccio, FedericaKawale, AjinkyaVeronese, FedericaKawano, KouichiroVeronesi, PaoloKawaoka, TomokazuVerrelle, PierreKawashima, MarikoVersari, AnnibaleKawauchi, KeikoVertegaal, AlfredKazanietz, Marcelo G.Verticchio, AliceKazlauskas, ArunasVerwaal, Victor JilbertKazuo, YashimaVesa, Stefan CristianKe, YiyuVeschi, SerenaKe, YouqiangVeschi, VeronicaKeek, SimonVeskoukis, Aristidis S.Keereweer, StijnVestal, Deborah J.Kefleyesus, AmanielVestergaard, Lau K.Keisari, YonaVetrano, Ignazio GaspareKeklikoglou, IoannaVezzani, BiancaKeller, Jonathan R.Vicario, NunzioKellner, ChristianVicent, SilvestreKelly, DervlaVider, JelenaKemeny, Nancy E.Vidovic, DejanKempiński, RadosławVidy, AuroreKenawy, NihalViel, AlessandraKendall, TimViel, SébastienKennedy, Michael A.Víglaský, ViktorKépénékian, VahanVignani, FrancescaKȩpka, LucynaVigneswaran, Wickii ThambiahKerbage, YohanVignoli, AlessiaKerkhofs, ThomasVigon, IsabelleKerkmeijer, LindaVijayasubhash, VinodKern, IzidorVilgrain, ValérieKern, PeterVillabona, LisaKerns, Jemma G.Villagomez-Bernabe, ‪BalderKersting, StephanVilla-Morales, MaríaKesh, KousikVillaverde, GonzaloKeshari, SunitaVillegas, VictoriaKesharwani, Siddharth S.Vincent-Salomon, AnneKessel, KatharinaVincenzo, RocchettiKessler, TorstenVinci, MariaKeszler, GergelyVinciguerra, ManlioKetola, KirsiVinti, LucianaKetteler, PetraViola, GiampietroKettenbach, JoachimViolet, Pierre ChristianKetter, RalfViossat, YannickKhairnar, Vishal S.Virolle, ThierryKhakoo, ShelizeVirumbrales-Muñoz, MaríaKhammanivong, AliVisani, GiuseppeKhan, Firdos AlamVisconti, Roberto MoroKhan, ImtiazVisentin, FabianoKhan, Mohd SajidVisone, RosaKhan, NilouferVisser, LydiaKhan, ShahanshahVitale, AlessandroKhan, ShaheenVitale, ChiaraKhare, PriyankaVitale, GiovanniKhazal, SajadVitale, MarioKhilov, Aleksandr V.Vitale, Rosa MariaKhivansara, VishalViti, VincenzaKhochenkov, DmitryVito, AlyssaKholová, IvanaViúdez, AntonioKhoury, ThaerVivas Mejia, PabloKhurana, NamrataVizler, CsabaKida, Yasuyuki S.Vizovišek, MatejKiechle, MarionVlahopoulos, SpirosKieler, MarkusVlašić, IgnacijaKiesel, LudwigVlassi, MetaxiaKiesewetter, BarbaraVleminckx, KrisKiewisz, JolantaVögeli, Thomas-AlexanderKikuyama, MasatakaVoisset, EdwigeKim, Byung SooVojtek, MartinKim, Chan GyooVolinia, StefanoKim, DonginVolkmer, BeateKim, Euishin EdmundVon Behren, JulieKim, Eun-HeeVon Bueren, André O.Kim, Hee SeungVon Der Grün, JensKim, Hee SungVon Eggeling, FerdinandKim, HongbeomVon Eyben, Finn EdlerKim, Hun SikVon Neuhoff, NilsKim, Hyeong-GeugVošmik, MilanKim, HyungjinVoulgarelis, MichaelKim, Ik-HwanVoutsinas, Gerassimos E.Kim, Jae-hwanVozenin, Marie-CatherineKim, JaesungVraka, ChrysoulaKim, Ja-EunVranic, SemirKim, Jin HyoungVrettos, NicholasKim, Jin-HongVriend, JerryKim, Jin-hwiVujanovic, NikolaKim, Jong ManVymetalkova, VeronikaKim, KibeomWada, KoichiroKim, Kyoung SubWade, RosKim, KyunggonWadell, GöranKim, KyunghwanWadham, CarolKim, Min-SikWagner, ArthurKim, Raymond H.Wagner, Kay-DietrichKim, Ryong NamWagner, Kay-UweKim, RyungsaWagner, LarsKim, Sang WunWagner, Michael J.Kim, Sang-WeWagner, RichardKim, Seok-JunWagner, SteffenKim, SoochiWagner, TristanKim, Su ZyWagner, WaldemarKim, Sun HwaWainman, AlanKim, Sung EunWakabayashi, YuichiKim, Sung JoonWakatsuki, MasaruKim, Sung-HoonWakimoto, HiroakiKim, Tae MinWałajtys-Rode, ElżbietaKim, Tae-DonWalczyk, AgnieszkaKim, UshaWald, Abigail I.Kim, WoojinWaldron, Richard T.Kim, Yeul HongWalker, BrianKim, Yong-MiWalker, Paul RaymondKimble-Hill, AnnWallin, GöranKimura, ShojiWalsh, NaomiKimura, YasutoshiWalter, FranziskaKimura, YoshiharuWalter, IngridKindt-Dunjko, AlidaWan, ThomasKing, Chih-YenWang, Cheng-HsuKing, ReneeWang, Cheng-YiKingsley, KarlWang, ChihyangKinj, RémyWang, ChunKino, KatsuhitoWang, DejieKinoshita, TomomariWang, GuannanKioi, MitomuWang, GuohaoKiouptsi, KlytaimnistraWang, HanwenKirac, IvaWang, Hee-JungKiran, SoniaWang, HeruiKirken, Robert A.Wang, HeshengKishore Sakharkar, MeenaWang, Hsiang-ChenKiss, NorbertWang, HuaishanKistenev, YuriiWang, HuanyouKitadate, AkihiroWang, JeffreyKitagawa, SeiichiWang, JiaoKitaichi, KiyoyukiWang, JiaxingKitano, MasayukiWang, Jing-QuanKitt, JayWang, JunfengKittel, AgnesWang, KaiKiyohara, EijiWang, LiangKlampatsa, AsteroWang, Lily Hui-ChingKlapdor, RüdigerWang, LishengKleeff, JörgWang, LuKleibl, ZdenekWang, Po-HuiKlein, AlexanderWang, ShidanKlein, SebastianWang, TingKleindienst, AndreaWang, WeiKleinman, HyndaWang, Yan-HsiungKlencki, MariuszWang, Yuan-HsiKlener, PavelWang, Yu-ChihKlepinin, AleksandrWanibuchi, HidekiKlepsch, VictoriaWaqar, MueezKlimaszewska-Wiśniewska, AnnaWard, DouglasKlimczak, AleksandraWard, Stephen C.Klimkowska, MonikaWarnakulasuriya, SamanKlinakis, ApostolosWarnken, ZacharyKlinge, Carolyn M.Watabe, TadashiKlink, MagdalenaWatanabe, EiichiKlinkhammer-Schalke, MonikaWatanabe, YoichiKluger, Harriet M.Watanabe, YutakaKlyui, Nickolai I.Wątek, MarzenaKnapper, StevenWaterfall, Joshua J.Knauf, JeffreyWaterton, JohnKnebel, CarolinWatson, SteveKnez, JureWatts, CarolineKnopfova, LuciaWebber, KateKnuschke, TorbenWeber, KlausKo, BeomseokWeber, ThomasKo, Jiunn- LiangWeber, WolfgangKo, MyunggonWebster, MarieKo, Na-ReWei, Hsiu-ChuanKo, RyoWei, JunKobaek-Larsen, MortenWei, Jun S.Kobayashi, HiroshiWei, LeiKobayashi, MakotoWei, YonglongKobayashi, NoritoshiWeichhaus, MichaelKobeissy, FirasWeinfeld, MichaelKoch, OliverWeis, SergeKochat, VeenaWeishaupt, CarstenKocian, RomanWeiss, NoelKodama, TakahiroWeiss, RobertKoehl, GudrunWei-Wu Chen, TomKoehl, Gudrun E.Welch, John S.Koelblinger, PeterWeldon, JohnKofler, LukasWelling, D. BradleyKoga, FumitakaWellner, Ulrich FriedrichKoga, YoshikatsuWelsh, MichaelKoh, DavidWendt, MichaelKoh, Yong QinWenger, KatharinaKoh, Young WhaWeniger, MaximilianKohyama, AtsushiWerner, AndreasKoivunen, JarkkoWertz, Philip W.Koivusalo, Antti IlmariWesołowska, OlgaKok, Chung HoowWessely, AnjaKokkonda, PraveenWestermarck, JukkaKolberg, Hans-ChristianWestphal, DanaKolekar, PandurangWhelan, Rebecca J.Kolesar, Jill MarieWhitehall, VickiKoliakos, GeorgeWibmer, Andreas G.Koljonen, VirveWickenhauser, ClaudiaKolkova, ZuzanaWidłak, PiotrKolligs, Frank T.Wieczfińska, JoannaKolomeǐchuk, SergeyWiedenmann, BertramKolot, Mikhail N.Wiegering, VerenaKolovskaya, OlgaWiemer, Erik A. C.Koma, YuichiroWierzejska, ReginaKomine, MayumiWiesenfeld, DavidKomohara, YoshihiroWieser, RotraudKomorowski, AndrzejWiesner, Georgia L.Komune, NoritakaWiggermann, PhilippKondo, MotonariWijaya, RameshKondo, TadakazuWiktorska, KatarzynaKondrashova, OlgaWilczek, BrigitteKong, Li RenWilczyński, Jacek R.Kong, MoonkyooWilhelm, ImolaKong, Siu-KaiWilhelmi, MarkusKonishi, HiroakiWilke, Christopher T.Konner, Jason A.Wilkens, LudwigKonoike, NahoWilkins, SimonKonsman, Jan PieterWilley, ChristopherKonstantakou, Eumorphia G.William, Tracy A.Konstantinos, TepetesWilliam, VermiKontogeorgos, GeorgeWilliams, Graeme P.Kontogianni, GeorgiaWilliams, Norman R.Kontos, Christos K.Wilm, MatthiasKoo, KendrickWilmink, HannekeKoo, Kevin M.Winer, Ira S.Koob, SebastianWinham, Stacey J.Kook, Myeong-CherylWinn, Deborah M.Koontz, Bridget F.Winter, ChristofKopaczyńska, MartaWinter, GordonKopec, MonikaWinter, StefanKopecký, JindrichWirscheim, IngridKopp, ReinhardWirsdörfer, FlorianKopp, SaschaWirth, MatthiasKoppisch, DorotheaWirth, UlrichKopsida, MariaWitalisz-Siepracka, AgnieszkaKorac Prlic, JelenaWitarski, WojciechKorać, AleksandraWitek, Malgorzata A.Korc, MurrayWitkowska Pilaszewicz, OlgaKordulewska, NataliaWitte, Hanno M.Korf, Horst WernerWlodawer, AlexanderKoritzinsky, MarianneWoessmann, WilhelmKornek, MiroslawWoitek, RamonaKorolkova, Olga Y.Wojewoda, MartaKorsching, EberhardWolach, OfirKorshoej, Anders RosendalWolf, BenjaminKosan, ChristianWolf, KatarinaKoseła-Paterczyk, Hanna M.Wollmann, GuidoKoshel, ElenaWołowiec, DariuszKostara, Christina E.Won, MisunKöster, ClausWong, Chi ChunKostev, KarelWong, Chi-MingKostic, Tatjana S.Wong, Kwan YeungKostopoulos, Spiros A.Woo, Chang-HoonKosuga, ToshiyukiWoo, ShiaoKosuge, YasuhiroWoo, SungminKot, KarolinaWood, KevinKota, SatyaWood, Lori AnneKotarski, JanWoods, Amina S.Kotaška, KarelWoodworth, Graeme F.Kotecha, Rupesh RajeshWorni, MathiasKotelevets, LarissaWorst, Thomas StefanKoukourakis, Michael I.Woźniak, MartaKouokam, J. CalvinWozny, Anne-SophieKouro, TakuWren, DörteKouroumalis, Elias A.Wright, Roni Helene GraceKoustas, EvangelosWrobel, Tomasz P.Kouttab, NickWróblewska, JoannaKovács, AttilaWrotek, SylwiaKovács, GyörgyWu, Alexander T. H.Kovalenko, Elena I.Wu, Anna H.Kowal, KrzysztofWu, CenKowalik, ArturWu, ChengyueKowalska, KarolinaWu, Chiao-EnKowalski, ChristophWu, Chun-TeKowarik, Markus C.Wu, ChunyiKoyama, TatsukiWu, DiKozlov, SergueiWu, JianmingKozłowska, AnnaWu, LicunKozono, DavidWu, Ming-HsunKozovska, ZuzanaWu, Ming-JiuanKrahn, ThomasWu, RigumulaKrainer, Michael W.Wu, TingKranc, WieslawaWu, WilliamKrane, L. SpencerWu, XiaohuaKranendonk, Mariëtte E. G.Wu, XuedanKranz, MathiasWu, Yi HuiKrarup-Hansen, AndersWu, YoujunKrasnodębski, MaciejWu, Yuan HuaKratzke, Robert ArthurWu, Yu-shanKrautz, ChristianWullaert, AndyKrawczyk, MalgorzataXavier, Sastre-GarauKrazinski, BartlomiejXia, BingKrebs, MarkusXia, JunKregždytė, RimaXiao, HuaKreipe, Hans H.Xiao, ZhoushengKreis, Nina-NaomiXie, ChangqingKreis, StephanieXie, GuofengKrengli, MarcoXie, Xian JinKress, AndreaXie, ZhiqunKretov, DmitryXie, ZhongqiuKrier, FrançoisXie, ZuoxuKrijgsman, DaniëlleXu, MeifengKrishnamachary, BalajiXu, Song-HuiKrishnan, ShanmugarajanXu, XiaoyinKristensen, Gunnar B.Xu, XuehuaKritsiligkou, ParaskeviXu, YanKroesen, MichielXue, FengKrokidis, MiltiadisYabe, MarikoKról, Sylwia KatarzynaYadav, DhananjayKron, PhilippYadav, PoonamKron, TomasYadav, SushmaKroneis, ThomasYagi, HiroshiKrönke, JanYahia, MassinissaKrug, SebastianYam, Jason C.Krum, Susan A.Yamada, HidetakaKŕušlin, B̧ožoYamada, SuguruKruyt, FrankYamada, TaketoKubo, NobuteruYamakawa, DaishiKucinska, MalgorzataYamamoto, MasahiroKudela, ErikYamamoto, NaokiKuehne, AndreYamamoto, YutakaKuemmel, SherkoYamamoto-Ibusuki, MutsukoKuhl, Christiane K.Yamamura, SoichiroKuhlmann, JanYamamura, TakeshiKuhn, ElisabettaYamanaka, RyuyaKühn, ThorstenYamao, KentarouKuittinen, OutiYamaoka, ToshimitsuKulbacka, JulitaYamasaki, SatoshiKulczyńska-Przybik, AgnieszkaYamasaki, TakahiroKulemzin, SergeyYamashita, HirokoKulesa-Mrowiecka, MałgorzataYamashita, KeishiKuliczkowski, KazimierzYamatsu, KojiKulka, JaninaYamauchi, AkiraKulkarni, DhananjayaYamazaki, TaigaKumar, A. PratapYammani, Raghunatha R.Kumar, AshishYan, ChunhongKumar, JananiYan, ShengminKumar, ManishYan, YuanweiKumar, PradeepYanagi, TerukiKumar, PrashantYáñez-Mo, MaríaKumar, RajYang, ChangwonKumar, RajeshYang, Chih-JenKumar, RajivYang, DonghanKumar, RavindraYang, Eun JooKumar, SandeepYang, HaifengKumar, SunilYang, HaitangKumar, VijayYang, Howard H.Kumarasamy, Vishnu MuthurajYang, Hsin-YiKumari, ShardaYang, Jiann-JouKumari, SnehlataYang, JiulingKumi-Diaka, JamesYang, JunKumta, Shekhar M.Yang, KaiKunda, NiteshYang, Lee-WeiKung, Hank F.Yang, Ming-HsinKung, Hsing-JienYang, QuansanKung, Hsiu-NiYang, SejungKunkler, IanYang, Seung-HoKuno, HirofumiYang, ShengyuKunstman, JohnYang, Sherry X.Künzel, JulianYang, Shun-FaKuo, Chao-HungYang, Wei-hsiungKuo, Chih-Hsi ScottYang, Wen-BinKuo, Ping-ChungYang, YaohuaKuo, Shu-JuiYang, YingKuo, Sung-HsinYang, YongkangKure, ShokoYang, Zeng-QuanKurmasheva, RaushanYang, ZhaogangKuroda, EtsushiYang, Zhiyu AmyKuroda, HiroakiYang-Hartwich, YangKuroda, JunyaYanguas-Casás, NataliaKurokawa, HiromiYao, RyojiKurokawa, TomohiroYao, XiaolanKuroyanagi, HiroyaYaoi, TakeshiKurth, JensYarravarapu, NageswariKuryk, LukaszYates, Luke A.Kušec, RajkoYe, Jing ChristineKushekhar, KushiYe, YunpengKusior, AnnaYe, ZhiweiKusumbe, AnjaliYeager, NicholasKutasovic, Jamie R.Yegorov, YegorKutler, DavidYeh, Chun-NanKutny, MatthewYeh, I-JuKutter, ClaudiaYeh, Kun-yunKuwahara, KazuhikoYeh, Ta-senKwack, KyuBumYélamos, JoséKwapiszewska, KarinaYeong, Joe Poh ShengKwee, SandiYeung, Tsz-LunKwok, Hoi-HinYevsa, TetyanaKwok, WaiYi, Hee-GyeongKwon, JaewooYi, Joo MiKwon, Jung HyunYi, TaoKwon, Seong YoungYin, KanhuaKwon, So HeeYing, MichaelKwong, JosephYlösmäki, ErkkoKypta, RobertYochum, Gregory S.Kyrgios, IoannisYoda, SatoshiKyriakou, IoannaYoh, TomoakiKyriazoglou, AnastasiosYohe, Marielle E.L. Balint, BalintYokobori, TakehikoLa Forgia, DanieleYokomichi, NaosukeLa Mantia, IgnazioYokota, HirokiLa Monica, SilviaYokota, TomoyaLa Rocca, Renato V.Yoneda, YukioLa Vaccara, VincenzoYong, WeiPengLa Verde, GiuseppeYoo, JeongsooLa, TingYoo, So YoungLaakmann, ElenaYoon, DonghoonLaboisse, Christian L.Yoon, HyonokLacina, LukasYoon, WonsuckLacombe, JeromeYoon, Won-SupLacour, BrigitteYoshida, DaisukeLadekarl, MortenYoshida, MasahitoLadetto, MarcoYoshida, NoriakiLaengle, JohannesYoshie, MikihiroLafont, ElodieYoshii, YukieLaganà, Antonio SimoneYoshinami, TetsuhiroLagopati, NefeliYoshino, HironoriLai, AndrewYoshino, KiyoshiLai, CatherineYoshio, SachiyoLai, Ching LungYoshioka, Ken-ichiLai, Chyong-HueyYoshioka, KentaroLai, FeipeiYost, Kathleen J.Lai, James J.You, XiaonaLai, Lu-HanYoun, Hyun JoLai, MicheleYounes, SherenLai, Ming-DergYoung, Christian D.Lai, RaymondYoung, Richard J.Lai, YanhaoYousefi, BardiaLaird, BarryYousefi, Behrooz HooshyarLajolo, CarloYousefzadeh, MatthewLakota, JanYu, Cheng-PingLalk, MichaelYu, Ching-FangLallena, María JoséYu, FangyanLalli, EnzoYu, HerbertLallous, NadaYu, Huang-PingLamarque, DominiqueYu, Jian Q.Lamarre, Eric D.Yu, JianhuaLamas Maceiras, MónicaYu, JunLambertini, MartinaYu, KaiLamfers, MartineYu, L.Lammers, ReinerYu, LingLampridis, SavvasYu, XiupingLan, Yuan-TzuYu, YanlinLandon, Chelsea DawnYu, YanpingLandreville, SolangeYu, Yung-LuenLandström, MaréneYuasa, MariLanduzzi, LorenaYue, JunmingLanfrancone, LuisaYuge, RyoLang, AlexanderYun, Seok JoongLang, FengchaoYunes, José AndrésLang, PeterYunlong, ChenLang, RolandZaboronok, AlexanderLangford, DaleZabucchi, GiulianoLanguino, LuciaZacchia, MiriamLannigan, DeborahZachos, GeorgeLanning, NathanZachos, IoannisLanuti, PaolaZachou, KalliopiLanza, FrancescoZaed, IsmailLanzi, CinziaZagożdżon, RadosławLanzillotta, MarcoZahid, MalihaLapidus, Rena S.Zahnreich, SebastianLapini, AlbertoZaidi, Syed H. E.Lapins, JanZaika, AlexanderLappano, RosamariaZaiss, DietmarLaranjo, MafaldaZaitone, Sawsan A.Lardone, Patricia J.Zakaria, RasheedLareau, CalebZakharenko, AlexandraLarese, FrancescaZakopoulou, RoubiniLarimer, Benjamin M.Zakrzewska, MalgorzataLarionov, Alexey A.Zalatnai, AttilaLarionova, IrinaZambelli, AlbertoLaRocque, JanZambello, RenatoLarsen, Anders ChristianZamboglou, ConstantinosLarue, LionelZambon, AlfonsoLasfar, AhmedZambrano, NicolaLastraioli, ElenaZamfir, Carmen LăcrămioaraLatham, MichaelZamò, AlbertoLatif, AyseZan, HongLatini, FrancescoZanelli, MagdaLatkauskas, TadasZanetti, RossellaLatosinsky, StevenZanetti-Domingues, Laura C.Latour, SylvainZani, Bianca MariaLatteri, SaverioZannoni, Gian FrancoLau, Andy T. Y.Zanzotto, Fabio MassimoLau, ChunghoZapico, Sara CasadoLau, DoreenZaporozhchenko, Ivan A.Laura, EvangelistaZapotoczny, GregoryLaurenzana, AnnaZaraca, FrancescoLaureys, GenevieveZarnegar, RasaLauth, MatthiasZarobkiewicz, MichałLautz, Timothy B.Zarovni, NatasaLavia, PatriziaZarrabi, KevinLawrence, WayneZarrine-Afsar, ArashLawrie, CharlesZattarin, EmmaLazarević, VladimirZavattari, PatriziaLazaris, AnthoulaZavattaro, ElisaLazennec, GwendalZawacka-Pankau, JoannaLazzarato, LorettaZebisch, ArminLazzeroni, MatteoZedenius, JanLe Bourhis, XuefenZeglis, BrianLe Romancer, MurielZehbe, IngeborgLe Roy, ChristineZehentmayr, FranzLe, Nguyen Quoc KhanhZeimet, Alain G.Lea, Michael A.Zeinali, MinaLeardini, DavideZelinka, TomasLebaron, RichardZemanova, ZuzanaLebbé, CélesteZemljic-Jokhadar, SpelaLeber, BettinaZemski-Berry, Karin A.Leboulleux, SophieZeng, BijunLeBrun, DavidZeng, JialiuLecis, DanieleZeng, YongjiLecka-Ambroziak, AgnieszkaŻeromski, JanLecuru, FabriceZhaler, StefanLederer, Eleanor DelandZhan, FenghuangLe-Deygen, I. M.Zhang, Casper J. P.Lee, AlexanderZhang, ChengchengLee, Byung ChulZhang, Daniel XinLee, Chang-HoonZhang, DongLee, Chang-minZhang, ErshuaiLee, Cheng-IZhang, GuangjunLee, Chia-HwaZhang, HonghaiLee, Chien-HsingZhang, HuanLee, Dae HoZhang, JiLee, DakeunZhang, JialingLee, Der-YenZhang, JianminLee, HoZhang, JileiLee, Hsueh-TeZhang, JinLee, Huei-JaneZhang, JingLee, HyunjuZhang, JixiangLee, Jae-HoZhang, LiLee, JaetaeZhang, LingLee, Jeng-changZhang, NuLee, JeongminZhang, PengfeiLee, Jeong-yeonZhang, QingLee, JieZhang, XiaotaoLee, Jih-ChinZhang, XiaowenLee, JiyoungZhang, XiaoyuLee, Joo YongZhang, YanfeiLee, Kang DaeZhang, YibinLee, KevinZhang, Yu-DongLee, Kin Wah TerenceZhang, YunLee, Kye YoungZhang, ZengdiLee, Mei-hwaZhang, ZhiguoLee, Seung-EunZhao, BailinLee, Shao-ChenZhao, DanLee, Soo-ChinZhao, LinboLee, Tsung-HsienZhao, WeixingLee, Tzong-ShyuanZhao, XiandaLee, Wei-HwaZhao, YongfengLee, Yen ChienZhao, YueLee, Yi-YenZhen, DavidLee, YongjunZheng, WenxinLee, ZhenghongZhong, XiaoboLeedham, SimonZhou, ChengjiLefebvre, Pauline M.Zhou, GangLeger, DavidZhou, JianLegler, Daniel F.Zhou, JingyingLehen′kyi, V′yacheslavZhou, QingyuLehmann, FredrikZhou, Tianhao (Vincent)Lehmann, Lorenz H.Zhou, WeihuaLehner, ManfredZhou, WenLei, WeiZhovannik, IvanLeila, KhojaZhu, ChrisLeiserowitz, Gary S.Zhu, DandanLejman, MonikaZhu, QuingLekka, MalgorzataZielinska, PaulinaLemay, GuyZielke, AndreasLemonnier, LoicZielonke, NadineLeng, RogerZifarelli, GiovanniLengyel, ZsuzsannaZimmer, JacquesLenzi, PaolaZimpfer, AnnetteLeon Mateos, LuisZimta, Alina-AndreeaLeon Serrano, JavierZingman, Leonid V.León, JosefaZironi, IsabellaLeoncini, LorenzoZivny, JanLeone, PatriziaZofall, MartinLeong, Sai MunZoia, CesareLepage, CecileZojer, NiklasLepik, Kirill V.Zong, Wei-XingLeppä, SirpaZonta, GiuliaLerch, Michael L. F.Zottel, AljaLesinski, GregoryZou, Wen-QuanLeslie, KimberlyZrnc, Tomislav A.Lesniak, WieslawaZsiros, EmeseLessel, DavorZsuzsanna, HevessyLeTourneau, ChristopheZubiaga, Ana MaríaLeung, DilysZubiri, LeyreLeung, JustinZucchetto, AntonellaLeung, KatherineZugaza, José LuisLeung, NelsonZúñiga, LeandroLeupold, JörgZuo, JianminLeutenegger, MarcelZupunski, LjubicaLev, SimaZwaan, C. MichelLevander, FredrikZwaenepoel, KarenLevi Sandri, Giovanni BattistaZwahlen, DanielLevine, HerbertZwergel, ClemensLevi-Polyachenko, NicoleZylicz, Zbigniew

